# Synchronized Photoluminescence and Electrical Mobility Enhancement in 2D WS_2_ through Sequence-Specific Chemical Passivation

**DOI:** 10.1021/jacs.4c11052

**Published:** 2024-12-11

**Authors:** Zhaojun Li, Henry Nameirakpam, Elin Berggren, Ulrich Noumbe, Takashi Kimura, Eito Asakura, Victor Gray, Deepa Thakur, Tomas Edvinsson, Andreas Lindblad, Makoto Kohda, Rafael B. Araujo, Akshay Rao, M. Venkata Kamalakar

**Affiliations:** † Solid State Physics, Department of Materials Science and Engineering, Uppsala University, 75103 Uppsala, Sweden; ‡ X-ray Photon Science, Department of Physics and Astronomy, Uppsala University, 75120 Uppsala, Sweden; § Cavendish Laboratory, University of Cambridge, JJ Thomson Avenue, Cambridge CB3 0HE, U.K.; ∥ Department of Materials Science, Tohoku University, Sendai 980-8579, Japan; ⊥ Physical Chemistry, Department of Chemistry-Ångström Laboratory, Uppsala University, 75120 Uppsala, Sweden

## Abstract

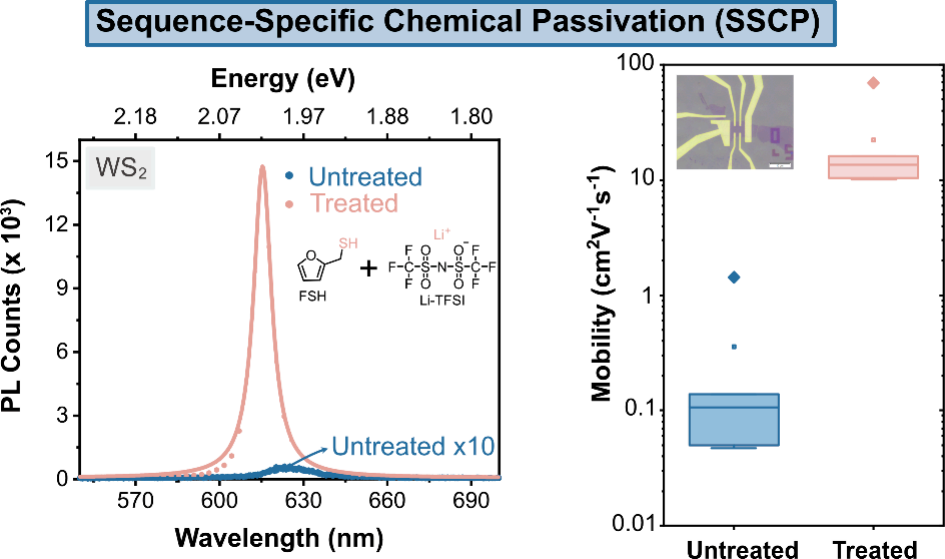

Two-dimensional (2D) semiconducting dichalcogenides hold exceptional promise for next-generation electronic and photonic devices. Despite this potential, the pervasive presence of defects in 2D dichalcogenides results in carrier mobility and photoluminescence (PL) that fall significantly short of theoretical predictions. Although defect passivation offers a potential solution, its effects have been inconsistent. This arises from the lack of chemical understanding of the surface chemistry of the 2D material. In this work, we uncover new binding chemistry using a sequence-specific chemical passivation (SSCP) protocol based on 2-furanmethanothiol (FSH) and bis(trifluoromethane) sulfonimide lithium salt (Li-TFSI), which demonstrates a synchronized 100-fold enhancement in both carrier mobility and PL in WS_2_ monolayers. We propose an atomic-level synergistic defect passivation mechanism of both neutral and charged sulfur vacancies (SVs), supported by ultrafast transient absorption spectroscopy (TA), Hard X-ray photoelectron spectroscopy (HAXPES), and density functional theory (DFT) calculations. Our results establish a new semiconductor quality benchmark for 2D WS_2_, paving the way for the development of sustainable 2D semiconductor technologies.

## Introduction

Atomically thin two-dimensional (2D) transition metal dichalcogenides (TMDs) have emerged as a new generation of semiconducting materials for electronic and optoelectronic applications.^1−5^ Photoluminescence (PL) intensity and charge carrier mobility (μ) are key indicators of the quality of 2D TMDs for optoelectronic applications as they are sensitive to traps, structural defects, and charged impurities.^6−8^ While offering extraordinary application potential, 2D TMDs face challenges from atomic defects such as chalcogenide vacancies, which trap charge carriers and induce nonradiative recombination pathways that quench carrier mobility and photoluminescence yield.^9−11^ Furthermore, the strong electrostatic interactions in 2D TMDs enable the formation of trions, quasiparticles composed of an exciton and free charges, even at room temperature.^12−15^ The existence of trions and defects strongly influences the intrinsic optical and electronic properties of the TMDs. Despite extensive research focused on growth and improving the semiconducting quality of 2D TMDs, the challenge remains in mitigating the defects within these materials.^16−23^ In this context, surface chemical strategies show promise as nondestructive methods to enhance the properties of 2D TMDs.^19,24^ However, the understanding of defects, particularly their interaction with passivating chemicals, remains unclear, which limits the progress in defect passivation.^25−27^ While conventional organic superacid H-TFSI results in trap-limited PL, damaging TMD materials and contacts and limiting its application in devices,^28,29^ other benign passivants, such as Li-TFSI, do not lead to electrical mobility improvement.^30^ Thus, no chemical treatment has yet significantly enhanced the PL and electrical mobility of 2D TMDs simultaneously, highlighting the need for innovative treatments that offer superior defect passivation and synergistically improve optical and electronic properties while remaining compatible with device fabrication.

In this work, by surface chemistry engineering of 2D WS_2_ monolayers, we innovate a sequence-specific chemical passivation (SSCP) protocoL using 2-furanmethanothiol (FSH, the key component of roasted coffee aroma) and bis(trifluoromethane) sulfonimide lithium salt (Li-TFSI). This chemical treatment protocol leads to simultaneous 100-fold enhancements in the charge carrier mobility and PL of mechanically exfoliated WS_2_ monolayers on SiO_2_ substrates, the highest enhancement factor observed with chemical passivation. The treatment also induces the largest blueshift in the PL peak position among all known surface treatments, signifying the most efficient p-doping effect and surpassing current benchmarks for the semiconducting quality of 2D WS_2_. In addition, these noncorrosive chemicals are stable and operate in benign solvents under ambient conditions, making them sustainable and suitable for direct use during the device fabrication of TMDs. The ultrafast transient absorption spectroscopy (TA) and Hard X-ray photoelectron spectroscopy (HAXPES) validate the high efficiency of the SSCP protocol and suggest a new interplay between chemical bonding and physisorption of defect-passivating agents, which is supported by our density functional theory (DFT) calculations. By identifying the defect nature and demonstrating how they can be effectively passivated for synergetic electrical and optical property enhancements, we enable the advancement of chemistry-based passivation techniques for 2D materials.

## Results and Discussion

**Figure 1:**
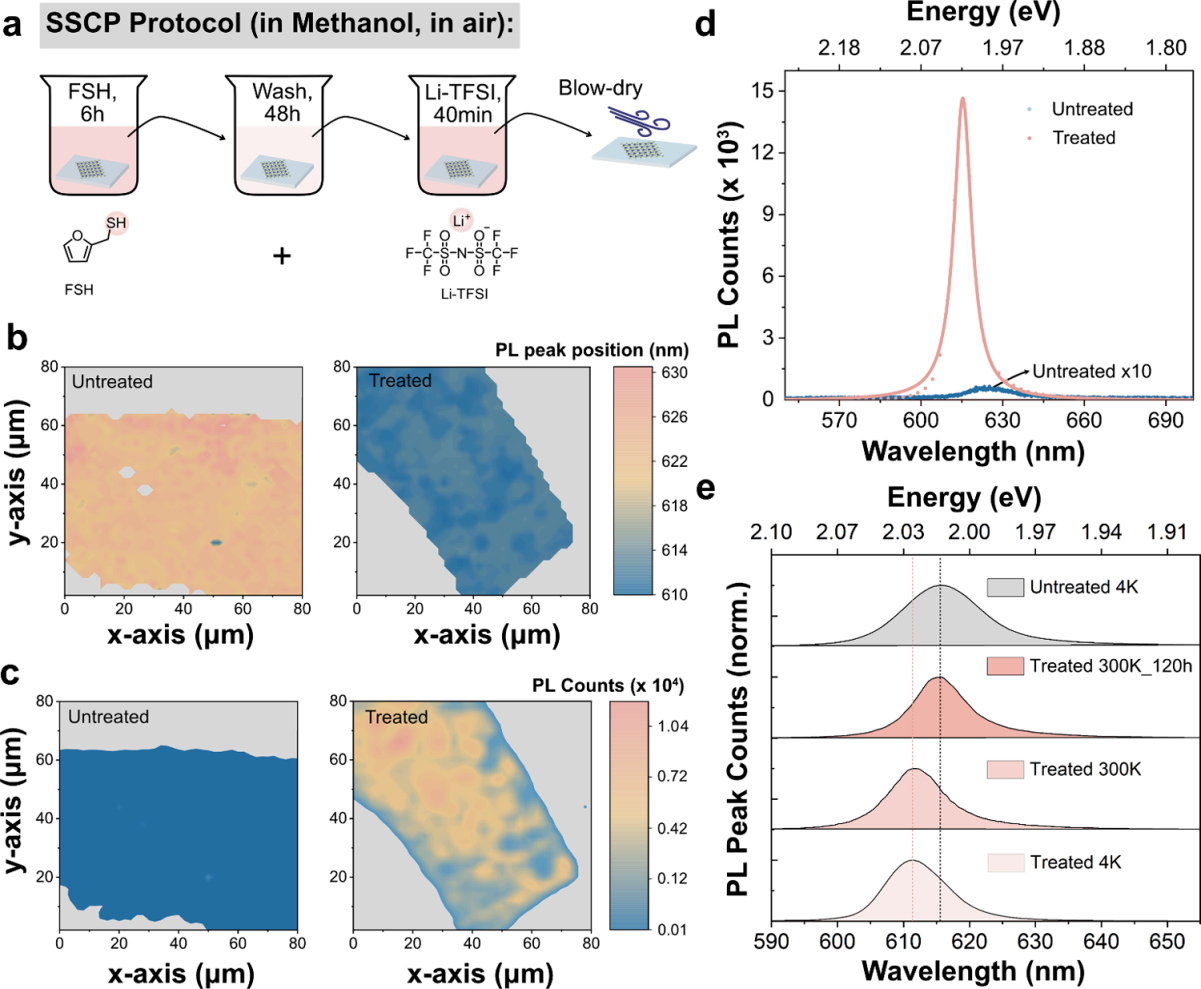
Illustration of the sequence-specific chemical passivation (SSCP) protocol and photoluminescence (PL) enhancement on monolayer WS_2_. (a) Illustration of the developed sustainable SSCP protocol procedure and the structures of the chemicals for the treatment (FSH + Li-TFSI). (b) Mapping of the PL peak position of untreated and treated samples. (c) Mapping of the PL intensity of untreated and treated samples. (d) Representative PL spectra for untreated and treated monolayer WS_2_. (e) Normalized PL spectra for the untreated and treated samples at 4 K compared with the normalized PL spectra for treated and stabilized treated sample (after treatment 120 h) at 300 K.

Our SSCP protocol for 2D WS_2_ monolayers (shown in Figure S1) integrates a thiol-based small molecule, FSH, and an ionic salt, Li-TFSI. The chemical structures and the optimized procedures of this protocol are depicted in Figure 1a. The FSH molecule consists of an electron-donating furan group, which increases its acidity and facilitates its solubility in alcohol-based green solvents. The hydrophilic salt Li-TFSI also presents a high solubility in alcohol-based solvents. The WS_2_ monolayer on a Si/SiO_2_ substrate was obtained by gold-assisted mechanical exfoliation, which provides larger monolayers that enable extensive characterization techniques that require large-scale 2D materials. The WS_2_ monolayer was immersed in a 0.01 M FSH/Methanol solution for 6 h. The extended duration ensures ample time for the FSH molecules to interact with the 2D WS_2_ surface. Following this, the sample was subjected to a cleansing process, where it was immersed in a Methanol solvent for 48 h. During this period, the solvent was replenished three times to ensure the removal of any excess FSH molecules that had not strongly interacted with the 2D surface. Finally, the sample was immersed in a 0.02 M Li-TFSI/Methanol solution for 40 min, after which it was air-dried without any additional washing steps. Notably, this stable chemical treatment protocol is developed in benign solvents and can be easily managed in an ambient atmosphere.

Following the SSCP protocol, the PL peak position exhibits a blueshift of over 30 meV throughout the entire monolayer flake, indicating that the macroscopic effect of this chemical treatment is p-doping (Figure 1b).^30−32^ Importantly, the PL of monolayer WS_2_ is greatly enhanced, increasing by up to 200 times, as shown in Figure 1c,d. The full width at half-maximum (fwhm) of the treated PL spectra is also reduced to 26 meV, compared to 64 meV for the untreated sample. The PL spectra profile of the 2D WS_2_ monolayer, measured immediately postchemical treatment at room temperature, aligns with that measured at 4 K in terms of the peak position and fwhm (Figures 1e and S2). This alignment suggests a significant suppression of defects and trions in the 2D WS_2_ following the SSCP.^33,34^ Given the PL inhomogeneity of the monolayer WS_2_ samples, we performed PL measurements on multiple WS_2_ monolayer samples. A comprehensive discussion on the statistical distribution of the PL peak position and the enhancement factor of the PL intensity with varied chemical treatments (Figures S3–S5) and the effect of the SSCP protocol on the WS_2_ monolayer with conventional Scotch tape (Figure S6) as well as the stability (Figure S7) is available in Supporting Information (SI) Note 4. The PL spectra of the treated sample underwent a redshift from 2.026 eV (612 nm) over time while stored in air, stabilizing at 2.019 eV (614 nm). This uniform change across the sample likely results from strain relaxation or doping from small molecules in the air.^22,35,36^

To understand the binding chemistry between chemicals and the 2D WS_2_ surface, we initially modified the chemical treatment procedures and evaluated the resultant changes in the PL spectra of 2D WS_2_ (Figures S8–S10). As shown in Figure S5, following the FSH treatment, we observe a homogeneous enhancement in the PL peak intensity across the monolayer flake of 2D WS_2_. This contrasts with the inhomogeneous shift in the PL peak position. (For a detailed discussion, refer to Supporting Information Note 4.) The subsequent treatment with Li-TFSI results in an inhomogeneous enhancement of PL intensity across the WS_2_ monolayer flake. This indicates that FSH and Li-TFSI interact differently with the WS_2_ surface, potentially passivating distinct types of defects.

Additionally, it is noteworthy that the PL peak position is robust to the washing process (Figure S6). After a 2 h immersion in methanol, the PL peak position and intensity remain stable across the monolayer flake (Figure S11, detailed discussion in Supporting Information Note 4). Even after 24 h of immersion, the PL peak position remains relatively stable despite a decrease in the PL intensity. This differential sensitivity of the PL peak position and intensity to the rinsing procedure suggests a complex interplay of factors governing these properties. Moreover, as depicted in Figures S7 and S8, the subsequent Li-TFSI treatment process reveals a gradual enhancement and blueshift in the emission of 2D WS_2_. Interestingly, this enhancement in PL intensity, induced by the Li-TFSI treatment, exhibits a level of reversibility, suggesting the absence of any chemical reactions between FSH and Li-TFSI, as well as between Li-TFSI and the surface of 2D WS_2_. Based on these observations, we hypothesize that the developed treatment protocol contributes to improved p-doping and alteration of the electronic structure of 2D WS_2_, leading to the observed furthest blueshift and largest intensity enhancement, respectively.

**Figure 2:**
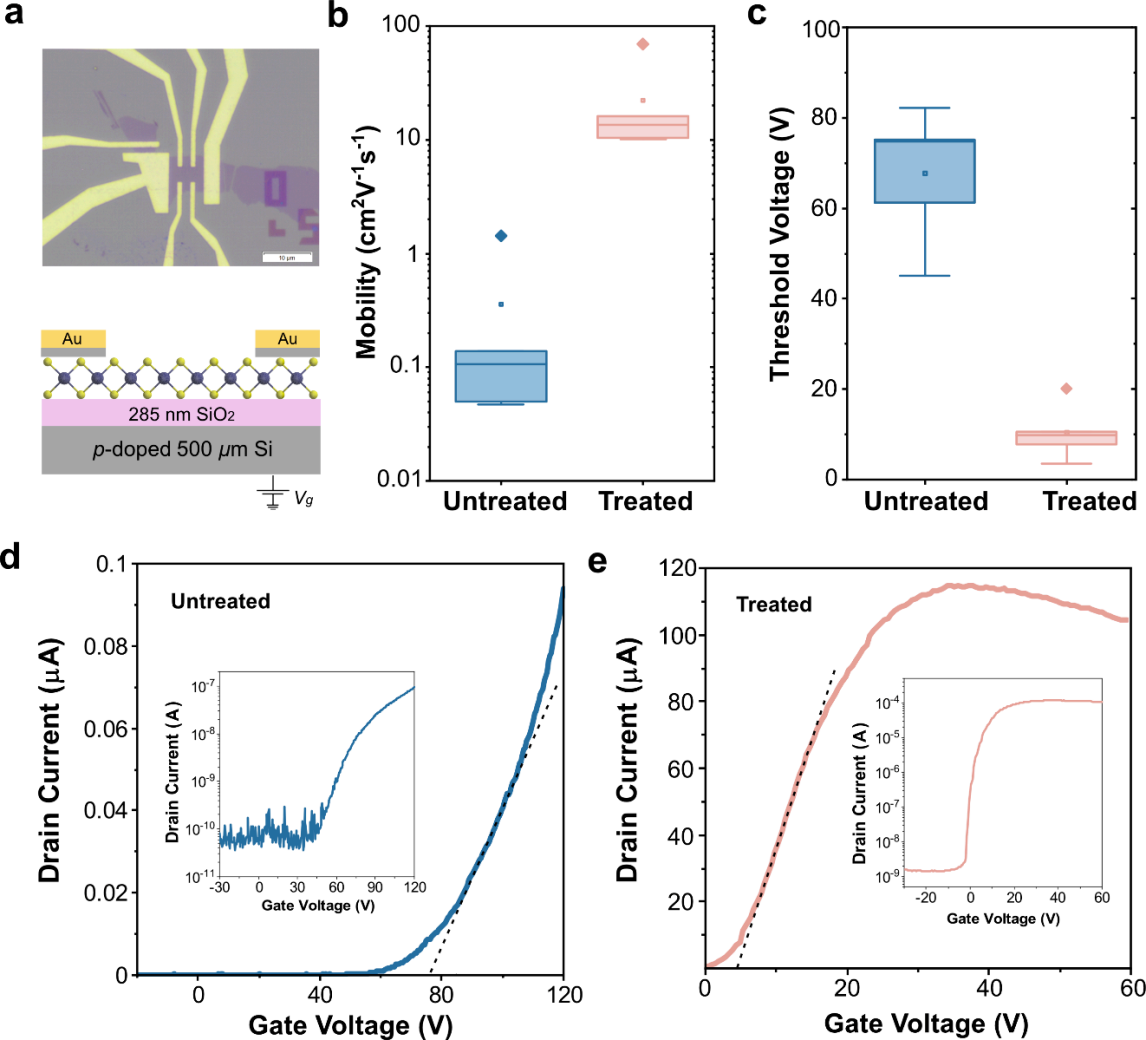
Electrical measurements at room temperature. (a) Optical image (top) and schematic representation (bottom) of an as-fabricated monolayer WS_2_ field-effect transistor (FET) device with Ti (5 nm) and Au (55 nm) contacts prepared using e-beam lithography patterning and electron beam metal evaporation. Scale bar: 10 μm. (b) Field-effect mobility for FET devices before and after treatment, plotting the mean (solid line) and standard deviation (box) on different devices. (c) Threshold voltage shift for FET devices before and after treatment. (d) Drain current versus gate voltage of untreated monolayer WS_2_ at drain-source voltage (*V*_DS_) of 1 V in linear scale. Inset: in logarithmic scale. (e) Drain current versus gate voltage of treated monolayer WS_2_ at *V*_DS_ of 1 V. Inset: in logarithmic scale.

Besides PL, charge-carrier mobility is a measure of semiconductor quality since it is very sensitive to impurities and traps. Here, we fabricated field-effect transistors (FETs) of WS_2_ monolayers and characterized the field-effect transport properties before and after treatment (Figure 2a). The electrical characterizations of transistors were conducted in a high-vacuum environment (∼10^–7^ mbar) to eliminate extrinsic doping and hysteresis effects induced by the adsorption of oxygen or water in the air. The detailed parameters for untreated and treated devices are presented in Supporting Information Note 5 (Tables S1 and S2 and Figures S12 and S13). The low field-effect mobility of untreated samples is in agreement with previous studies. As depicted in Figure 2b, we observe a striking two-order increase in the field-effect mobility, reaching 70 cm^2^ V^–1^ s^–1^ at room temperature. This is the highest value achieved for the WS_2_ monolayer on SiO_2_ substrates without encapsulation or introduction of a high κ dielectric environment at room temperature. Such an enhancement can be attributed to the passivation of the sulfur vacancy sites, which is expected to reduce the long-range coulomb scattering. This could also explain the observed increase in OFF current in Figure 2d,e, as well as Figures S12 and S13. The slight decrease in ON current at higher voltages in these figures might be due to increased Li^+^ ion scattering at high gate voltages where no more available states exist. The presence of Li^+^ ions may create an electrostatic disorder near the conducting channel, leading to reduced conductivity.^37^ The treated FETs exhibit an average field-effect charge mobility of ∼12 cm^2^ V^–1^ s^–1^, while that of untreated FETs is ∼0.1 cm^2^ V^–1^ s^–1^. At the same time, we also observe small variations that can originate from different channel lengths.^38^

In addition to the two-order enhancement in mobility upon passivation, we observe a clear decrease in the threshold voltage (*V*_T_) after the chemical treatment (Figure 2c). The *V*_T_ is extracted using the extrapolated linear region (ELR) method, as illustrated in Figure 2d, e. Statistically, the *V*_T_ values for the treated transistors were found to be 12 ± 8 V, while the untreated transistors exhibited a larger variation, with values lying in the range of 64 ± 19 V. This shift can be attributed to the p-doping effect from the Li^+^ ion, which is expected to decrease the concentration of trions, thereby facilitating easier charge transfer across source and drain terminals. This Fermi-level shift in 2D WS_2_ aligns well with the PL measurements discussed above and the DFT simulation discussed later. Notably, our SSCP protocol leads to a three-order-of-magnitude decrease in the total FET resistance. The electrical measurements suggest a synergistic effect of increased doping and sulfur vacancy defect passivation due to the SSCP.

**Figure 3:**
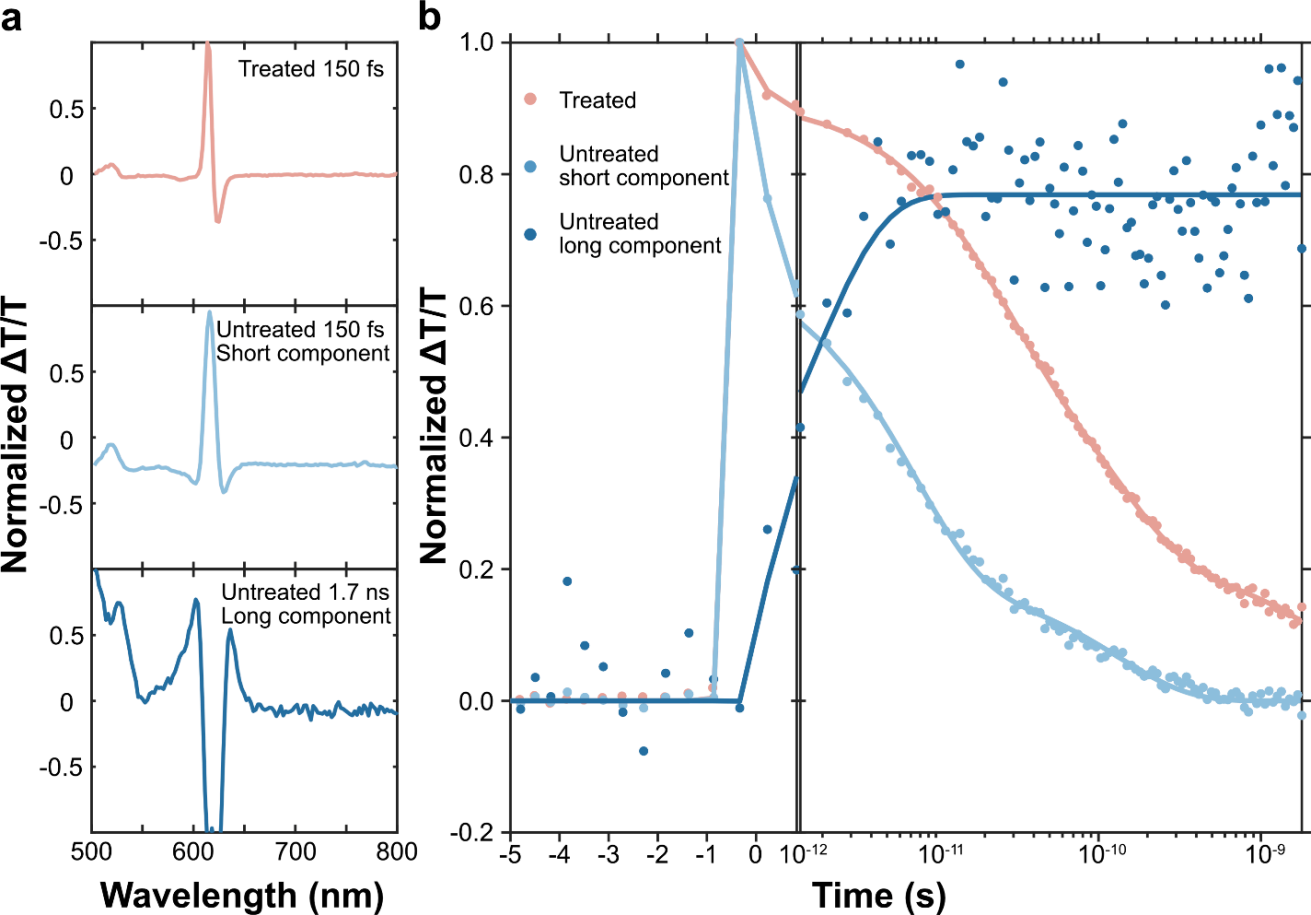
Pump–probe spectra and excited-state dynamics of untreated and treated monolayer WS_2_. (a) Normalized pump–probe spectra of treated and untreated monolayer WS_2_ at 150 fs and normalized pump–probe spectrum at 1.7 ns for untreated monolayer WS_2_. (b) Kinetic profiles for the corresponding spectra in a and multiexponential fits using a 185 fs-wide Gaussian response function. The fitting is presented in solid lines.

To further understand the effect of chemical treatment on the optical and electronic properties, we studied the WS_2_ monolayers by using fs-transient absorption (fs-TA). It measures the change in transmission after the sample is excited by a ∼200 fs laser pulse. In our experiments, a positive  signal corresponds to when the excited sample transmits more light due to the depopulation of the ground state, referred to as ground-state bleach (GSB). Negative  signals can arise due to the absorption of an excited state (photoinduced absorption, PIA) or the stimulated emission (SE) from the excited state. We compared multiple untreated monolayers to understand the sample variations (Figure S14). All samples show similar spectral features and only minor differences in the decay dynamics. It is clear that for the untreated samples, two spectral components are observed. This is also supported by single value decomposition (SVD) of the experimental data set, where two components show significantly larger singular values and spectral components above the noise level (detail in Supporting Note 6, Figures S15 and S16). From the TA data, we can identify an initial spectrum (100 fs–1 ps) with a positive peak corresponding to the A-exciton GSB at 616 nm (Figures 3 and S17) and another peak from the B-exciton GSB at 520 nm. Over the first 10 ps, these features disappear, and a broad positive feature between 600 and 650 nm arises with a sharp negative peak overlapping at 620 nm. The dynamics of the spectral components are extracted using spectra at early (150 fs) and long (1.7 ns) times, as detailed in Supporting Note 6. The initial component exhibits an average lifetime of 1–5 ps across two independent samples, with the emergence of a broad feature occurring concurrently. The broad feature is similar to that observed in liquid-exfoliated WS_2_ in our previous work.^39^ We previously assigned this broad feature to the GSB of multilayer WS_2_ arising from the energy transfer. However, as the prepared mechanically exfoliated samples here do not have any multilayer parts (Figure S1c), this feature must be associated with something else. We assign this to the GSB of a charged sulfur vacancy trap state, which is supported by our DFT simulation, as we discuss subsequently.

Interestingly, comparing the trap-state feature signal at 1.7 ns reveals a relatively minor dependence on the intensity. In Figure S14, we compare the signal between low (50 W) and high (360 W) excitation. Despite a more than 7-fold increase in excitation intensity, the initial GSB increases proportionally, while the trap-state signal only doubles. This suggests a limited number of traps that can be populated from the initial excited state. A similar observation is made when the excitation is changed from 610 to 510 nm, where the sample’s higher absorption leads to a greater initial excited-state population and GSB signal. However, the trap-state signal remains consistent with that observed with 610 nm of excitation. Following the SSCP protocol, the initial GSB signal experiences a slight blueshift to 614 nm, with only one spectral component observed (refer to Figures 3 and S18). Simultaneously, the GSB signal decays with an average lifetime of 31 ps, an order of magnitude slower than that of the untreated samples. These observations collectively provide clear evidence that the SSCP protocol developed in this study effectively passivates (removes) the sulfur vacancy trap states in 2D WS_2_, thereby extending the excited-state lifetime and slowing the decay dynamics. This explains the observed increases in PL photon intensity and charge carrier mobility.

**Figure 4:**
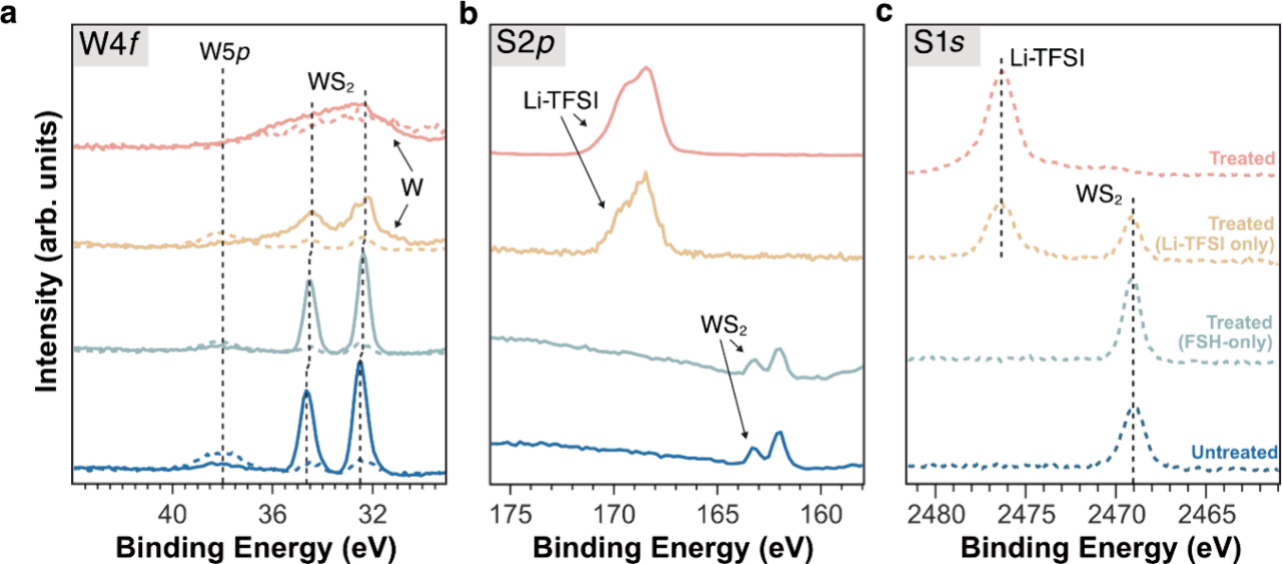
X-ray photoelectron spectroscopy (XPS) and hard X-ray photoelectron spectroscopy (HAXPES) measurements. Core levels of (a) W 4f, (b) S 2p, and (c) S 1s before and after surface treatments. Spectra displayed with dotted lines are measured with HAXPES.

To determine the stoichiometry of untreated and treated WS_2_ monolayers, we employed XPS and HAXPES. The W4*f* and S2*p* core-level photoelectron spectra were analyzed, and the ratio of the two core-level areas was compared before and after surface treatment (Figure 4). The area of the respective peak was obtained from the fit after background subtraction using curve fitting. The Scofield ionization cross-section values for the respective core levels were taken into account (1.68 for S2*p* and 9.80 for W4*f*).^40^ The S/W ratio was 1.81 for the untreated WS_2_ sample and 2.43 for the sample treated with FSH. The increase in the relative amount of sulfur suggests that sulfur from the FSH surface treatment fills some of the SVs in the WS_2_ monolayer.

The sulfur 2*p* core-level spectra, as depicted in Figure 4b, reveal a WS_2_ binding energy of 162.0 eV (2*p*_3/2_ component) for both untreated WS_2_ and the FSH-treated sample. In the case of treated samples and samples treated with Li-TFSI only, sulfur peaks originating from Li-TFSI are observed. These peaks exhibit similar binding energies, with a minor shift of 0.1 eV between the two samples (2p_3/2_ component at 168.4 and 168.3 eV, respectively), indicating no chemical reaction with Li-TFSI treatment.^41,42^ This is further supported by the HAXPES measurement showing that The S 1*s* peak originating from the WS_2_ layer (2470.1 eV) is consistent across the untreated WS_2_, the FSH-treated, and the Li-TFSI-only treated sample (Figure 4c). The addition of metallic tungsten is observed in both the Li-TFSI and FSH + Li-TFSI-treated samples at relatively higher levels in the former. The formation of metallic tungsten was previously attributed to damages to the WS_2_ monolayer caused by sputter cleaning with Ar^+^ ions.^43^ Given that the experimental conditions are identical for all untreated and treated samples and that this metallic feature appears exclusively in the Li-TFSI and FSH + Li-TFSI-treated samples (with a higher percentage in the latter treatment), this strongly suggests that the presence of metallic tungsten is due to Li–W ionic bonding. A similar broadening of the W 4f peak was previously observed for the hydrogenation of the WO_3_ film.^44^ The large size and high quality of 2D material prepared in our work enable the observation of the Li–W ionic bonding, which was not rationalized before. The overall XPS spectra of WS_2_ before and after varied treatments with detailed discussion can be found in Supporting Note 7, Figure S19, and the core-level binding energies are summarized in Table S4.

**Figure 5:**
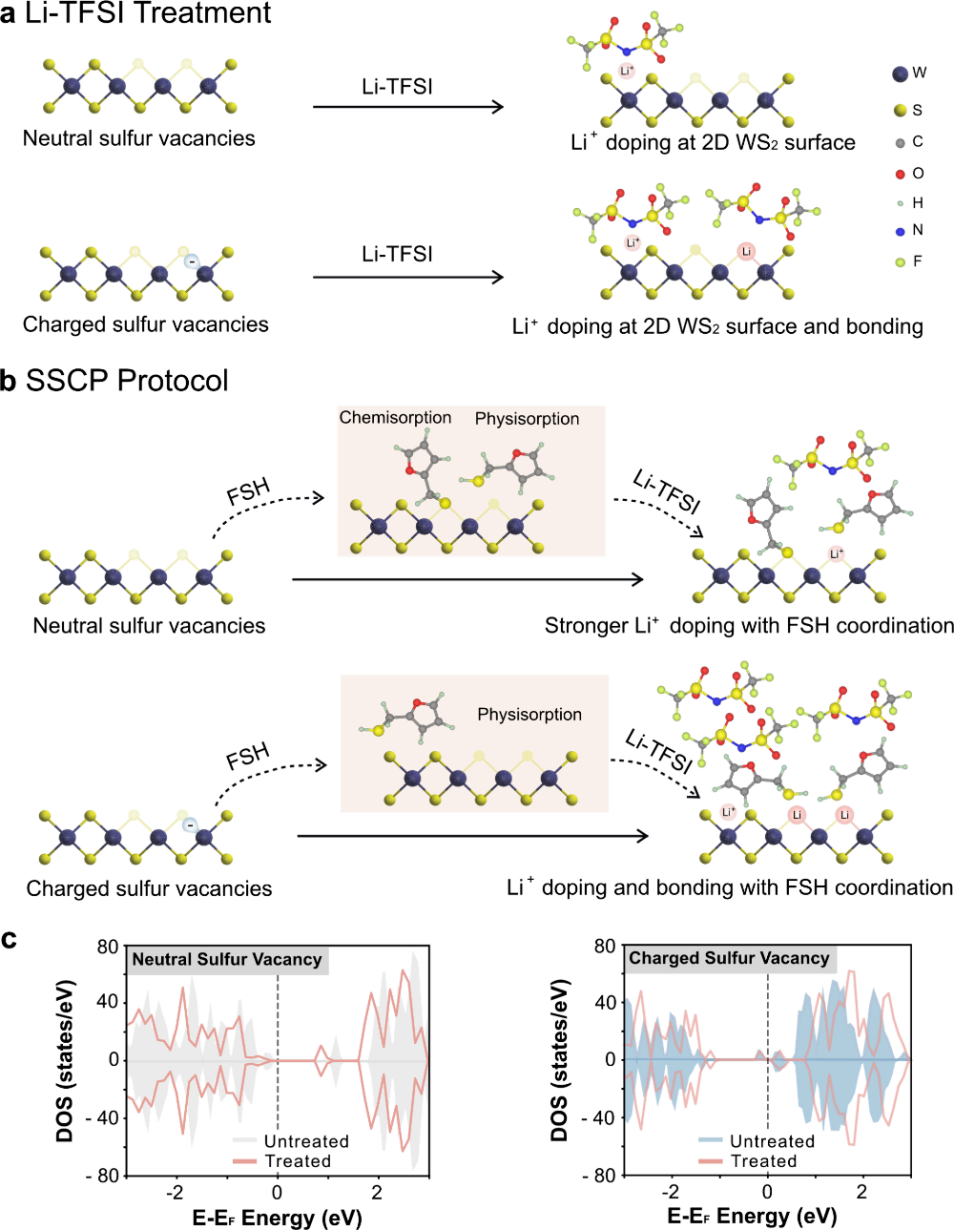
Illustration of the surface chemistry of 2D WS_2_during chemical treatments. (a) Schematic picture of the surface chemistry of 2D WS_2_ during the Li-TFSI alone treatment. The structure used in DFT calculations can be found in Supporting Information Note 8. (b) Schematic picture of the surface chemistry of 2D WS_2_ during the SSCP protocol. (c) Summed projected density of states (PDOS) on the 2D WS_2_ layers before and after the SSCP, while a neutral SV is considered (left) and a charged SV is considered (right).

To obtain insights into the mechanisms of SVs passivation on the WS_2_ monolayers by synergetic chemical treatment, we performed ab initio density functional theory (DFT) calculations to gain insights into the mechanisms of sulfur vacancies (SVs) passivation on the WS_2_ monolayers by synergetic chemical treatment. Summed PDOS calculations on atoms of the 2D layer were performed for various scenarios, encompassing the presence of a cation (Li^+^), anion (TFSI^–^), and physisorbed thiols on top of the WS_2_ monolayer. Both neutral (Figure S20) and negatively charged SV defects (Figure S21) were considered in these scenarios. Bader analysis was employed to deduce the insertion or removal of electrons within the layer. Our DFT calculations reveal that the FSH molecule effectively saturates the 2D WS_2_ surface, thereby reducing the presence of the solvent methanol due to their strong van der Waals interactions with the WS_2_ monolayer. Depending on the local environment, when a thiol group attaches to the monolayer surface, the S–H bond can cleave. This initiates chemical absorption of the remaining S onto neutral SV defects, which alters the electronic structure of the 2D layer, resulting in shallower defective states. (Figure S22). The sulfur absorption process is less likely to occur in negatively charged SV defects due to lower adsorption energies between the cleaved molecule and the negatively charged defect. Following treatment with Li-TFSI, lithium cation (Li^+^) stably adsorbs onto both the negatively charged defects and neutral defects (Figure 5c and Videos S1 and S2). Notably, for the neutral SV defect, changes in the summed PDOS of the layer were observed exclusively when FSH coordinated with Li^+^ (Figure 5a,b). By comparing with the TA data, the subgap of untreated 2D WS_2_ is closer to the band edge, which is responsible for the broader absorption feature in the TA measurements. Li-TFSI treatment shifts the band gap for negatively charged SV away from the conduction band edge, making the repopulation of charges from the trap state unlikely. Furthermore, Bader charge analysis revealed a reduction of 0.11 electrons for Li^+^ adsorption in the negatively charged SV defect, while in the scenario involving neutral SV defects, 0.08 fewer electrons were observed when Li^+^ was coordinated by the FSH molecule. The calculations do not reveal any electron insertion or removal from the layer in other cases (detailed discussion in Supporting Information Note 8, Table S5, Figure S23).

Overall, the neutral SV defects are likely to be populated by cleaved FSH molecules with S atoms and Li^+^ adsorption coordinated by FSH. In contrast, for negatively charged SV defects, FSH-coordinated Li^+^ adsorption dominates. Our study provides a distinct atomic-level synergistic defect passivation mechanism and rationalizes the nature of the defects: neutral and charged sulfur vacancies. While the two sulfur vacancies may appear similar, they necessitate distinct chemical passivation procedures. Superior optical and electrical performance can be achieved only by appropriately passivating both defect types.

## Conclusions

Through an innovative Sequence-Specific Chemical Passivation (SSCP) protocol, we demonstrate a synchronized two-order enhancement in carrier mobility and PL in WS_2_ monolayers. A 200-fold enhancement in PL is characterized by a narrower fwhm and a significant blueshift in the PL peak position, aligned with low-temperature PL measurements that indicate intrinsic PL behavior. Simultaneously, we observed a 100-fold increase in charge mobility at room temperature, reaching values up to 70 cm^2^ V^–1^ s^–1^ at room temperature. Our large-area WS_2_ monolayer samples have enabled comprehensive characterizations, including ultrafast transient absorption spectroscopy and X-ray photoelectron spectroscopy (XPS), which unequivocally confirmed the effectiveness of defect passivation. Notably, XPS shows the Li–W bonding, supporting our distinct defect passivation mechanism, which was not rationalized before. Supported by DFT calculations, our work provides an atomic-level chemical understanding of surface chemistry, detailing a distinct interplay between chemical bonding and the physisorption of defect-passivating agents that leads to observed synergetic enhancements. Our work provides a framework for binding chemistry engineering of 2D WS_2_ via the sustainable SSCP protocol for advancements in 2D electronic and optoelectronic applications.

## Methods

### Material and Sample Preparation

Bulk WS_2_ crystals were purchased from 2D Semiconductors. The monolayer WS_2_ was prepared according to the reported gold-mediated exfoliation method to ensure large monolayers.^45^ In this study, all experiments were carried out on monolayers. All chemicals for the surface treatments were purchased from Sigma-Aldrich and used as received.

### Spectroscopic Characterization

The temperature-dependent PL measurement is performed using an excitation wavelength of 544 nm, excitation power of 150 μW, and integral time 1 s for untreated and H-TFSI-only treated samples. Excitation wavelength 561 nm, excitation power 150 μW, and integral time 1 s are used for the treated sample. The microscope steady-state PL measurement was carried out using a WITec alpha 300 s setup and has been described previously.^46^ Importantly, a 405 nm continuous wave laser (Coherent CUBE) was used as the excitation source. A long pass filter with a cutoff wavelength of 450 mm was fitted before signal collection to block excitation scatter. The light was coupled with an optical fiber to the microscope and focused using a 20× Olympus lens. Samples were placed on an X-Y piezo stage of the microscope. The PL signal was collected in reflection mode with the same 20× objective and detected using a Princeton Instruments SP-2300i spectrometer fitted with an Andor iDus 401 CCD detector. The PL maps were measured with 405 nm excitation with a fluence of 15 W cm^–2^. The Raman measurements were carried out using a Renishaw inVia Raman confocal microscope with a 532 nm excitation source. Transient absorption was performed on a setup described previously.^47^ Details can be found in Supporting Note 1. The X-ray Photoelectron Spectroscopy (XPS) measurement was employed using an Al–Kα radiation source at a photon energy of 1486.6 eV. For the HAXPES measurement, a Ga–Kα radiation source at 9252.8 eV photon energy was used. Details can be found in Supporting Note 1.

### FET Device Fabrication and Measurements

The monolayer WS_2_ flakes were mechanically exfoliated using the gold-mediated exfoliation method and transferred directly on top of a highly p-doped Si/SiO_2_ (285 nm) substrate initially patterned with a network of alignment marks. The marks at the corners of the substrates are protected by the photoresist. Following up, the gold was etched in a solution of potassium monoiodide, and the substrate was rinsed in isopropyl alcohol (IPA). The flakes were then identified by optical contrast, and the source-drain electrodes were patterned by electron beam lithography. Prior to exposure, we spin-coated a bilayer-positive resist of MMA EL 9 and ARP 6200.13 on the substrate. The exposed pattern was developed using timed steps of hexyl acetate, methyl isobutyl ketone (MIBK)/IPA, and IPA. Following that, we evaporated 5 nm of the Ti seeding layer and 55 nm of Au in a high-vacuum chamber with e-beam evaporation. The fabrication was concluded with a lift-off in hot acetone at 70 °C for 10 min, rinsing in IPA at room temperature for 5 min and drying with nitrogen gas. Room-temperature electrical measurements were carried out in a high-vacuum cryostat (∼10^–7^ mbar) cryostat using an SMU K2450 to control the back-gate voltage and source meter K2400 for source-drain bias.

### Theoretical Calculations

The density functional theory (DFT) calculations were performed using the Projected Augmented Wave (PAW) method to solve the Kohn–Sham equations as implemented in the Vienna Ab initio Simulation Package (VASP).^48,49^ The spin-polarized generalized gradient approximation has been used with the Perdew, Burke, and Ernzerhof (PBE) parametrization to describe the exchange and correlation term of the Kohn–Sham Hamiltonian.^50^ Moreover, the DFT+D3 approach was used to take into account the van der Waals interactions.^51,52^ Details can be found in Supporting Note 2.

## Supporting Information Available

Neutral defects (MP4)
Charged defects (MP4)
Additional experimental and calculation details as well as additional data for optical and electrical characterizations of 2D WS_2_ samples and DFT simulations (PDF)
