# Supplementary material for: Synchronized Photoluminescence and Electrical Mobility Enhancement in 2D WS2 through Sequence-Specific Chemical Passivation
Source: J Am Chem Soc. 2024 Dec 11;146(51):35146–54. doi: 10.1021/jacs.4c11052 (PMC11673568; doi:10.1021/jacs.4c11052)
Supplement: Supplementary file 3 — ja4c11052_si_003.pdf [file ja4c11052_si_003.pdf]

Supporting Information

Synchronized Photoluminescence and Electrical Mobility Enhancement in 2D WS_2 through Sequence-Specific Chemical Passivation

Zhaojun Li[1,2,3]*, Henry Nameirakpam[2], Elin Berggren[2], Ulrich Noumbe[2], Takashi Kimura[4], Eito Asakura[4], Victor Gray[5], Deepa Thakur[1], Tomas Edvinsson[1], Andreas Lindblad[2], Makoto Kohda[4], Rafael B. Araujo[1]*, Akshay Rao[3]*, M. Venkata Kamalakar[2]*

[1]Solid State Physics, Department of Materials Science and Engineering, Uppsala University, 75103 Uppsala, Sweden.
[2]X-ray Photon Science, Department of Physics and Astronomy, Uppsala University, 75120 Uppsala, Sweden.
[3]Cavendish Laboratory, University of Cambridge, JJ Thomson Avenue, CB3 0HE, Cambridge, United Kingdom
[4]Department of Materials Science, Tohoku University, Sendai 980-8579, Japan.
[5]Physical Chemistry, Department of Chemistry-Ångström Laboratory, Uppsala University, 75120 Uppsala, Sweden.
*Email: zhaojun.li@angstrom.uu.se; rafael.araujo@angstrom.uu.se; ar525@cam.ac.uk; venkata.mutta@physics.uu.se

Contents

1. **Supplementary Note 1 - experimental details** 2
2. **Supplementary Note 2 - calculation details** 2
3. **Supplementary Note 3 – Material characterization** 3
4. **Supplementary Note 4 - PL data for chemically treated monolayer WS_2** 3
5. **Supplementary Note 5 – FET device data** 11
6. **Supplementary Note 6 – Transient absorption spectroscopy data** 15
7. **Supplementary Note 7 – Photoelectron spectroscopy data** 21
8. **Supplementary Note 8 – DFT data** 24
9. **References** 31

1. Supplementary Note 1 - experimental details

Si-SiO_2 substrates with 90 nm oxide layer were used for steady-state photoluminescence (PL), Raman spectroscopy and X-ray photoemission spectroscopy (XPS). Quartz substrates were used for ultrafast pump-probe measurement. All the measurements are carried out on samples without encapsulation.

Transient Absorption Spectroscopy measurement. A Light Conversion PHAROS laser system with 400 μJ per pulse at 1030 nm with a repetition rate of 38 kHz was used and the output was divided, one part was focused onto a 4 mm YAG substrate to produce a continuum probe beam from 520 to 950 nm. The second part of the PHAROS output was led into a narrow band optical parametric oscillator system (ORPHEUS-LYRA, Light conversion) outputting the pump beam. The probe pulse was delayed up to 2 ns with a mechanical delay stage (Newport). A mechanical chopper (Thorlabs) was used to create an on-off pump-probe pulse series. The pump size on the sample is approximately 0.045 mm^2 and the probe is about 0.008 mm^2. A silicon line scan camera (JAI SW-2000M-CL-80) fitted onto a visible spectrograph (Andor Solis, Shamrock) was used to record the transmitted probe light. The probe beam had a diameter of approximately half of the mechanically exfoliated monolayer size. Using a pin-hole to locate the monolayer the samples were placed to maximize the initial ground state bleach signal, ensuring that the probe was only probing the monolayer region. The pump was significantly larger than the monolayer to ensure uniform excitation across the whole monolayer. All obtained data was background and chirp corrected before analysis.

The X-ray Photoelectron Spectroscopy (XPS) measurement. Core levels and overview spectra were collected using a Scienta Omicron EW4000 hemispherical electron analyzer with a slit of 500 mm and a 200 eV pass energy. Binding energy calibration was obtained by aligning the Fermi level to a value of half the bandgap of monolayer WS_2, reported at 2.15 eV.[1] The experimental energy resolution was determined by the least squares fit of Au4*f* spectra using Voigt functions, employing 0.28 eV Au4$f_{5/2}$ and 0.3 eV Au4$f_{7/2}$ lifetime widths.[2] For XPS measurements this gave a Gaussian width of 0.62 eV and for HAXPES measurements at 0.88 eV. The Igor Pro 7.08 software and a CurveFitting procedure were employed to analyze the core level spectra [Edwin Kukk. Spectrum Analysis for Curve Fitting (SPANCF) macro package for Igor Pro.]. Voigt functions were utilized to fit the core level peaks, while the inelastic background was modeled using a Shirley function.[3]

2. Supplementary Note 2 - calculation details

The density functional theory (DFT) calculations. The density functional theory (DFT) calculations were performed using the Projected Augmented Wave (PAW) method to solve the Kohn-Shan equations as implemented in the Vienna Ab initio Simulation Package (VASP).[4,5] The spin-polarized generalized gradient approximation has been used with the Perdew, Burke, and Ernzerhof (PBE) parametrization to describe the exchange and correlation term of the Kohn-Sham Hamiltonian.[6] Moreover, the DFT+D3 approach was used to take into account the

van der Waals interactions.[7,8] Plane waves were expanded to an energy cut-off of 520 eV while Γ point was used to sample the Brillouin zone. Here, it is important to emphasize that the lattice parameters of the employed supercells are a = 15.9 Å and b=15.9 Å with a vacuum of 20.0 Å to avoid periodic interaction between the layers. Forces convergence was set to 0.01 eV/Å while an energy convergence to 10^{-5} eV. Gibbs free energy variation of the adsorption energies was calculated as $\Delta G = \Delta E + \Delta ZPE + (\Delta H_{vib} + \Delta H_{rot} + \Delta H_{trans}) - T(\Delta S_{vib} + \Delta S_{rot} + \Delta S_{trans})$ where ΔZPE is the variation on the zero-point energy, ΔH and ΔS are the variations of enthalpy and entropy, respectively, and ΔE is the electronic energy change. Here, the final state is assumed to be the molecule + slab, while the initial state is the slab + molecule in the gas phase. For the gas-phase calculations, the molecules were placed in a box with a lattice parameter of 25 Å to avoid interaction between periodic images. Vibrational frequency calculations were performed using a finite difference approach.

3. Supplementary Note 3 – Material characterization

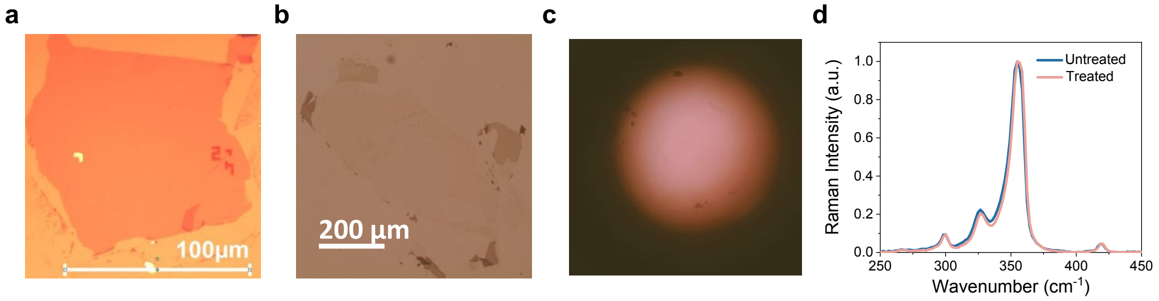

Supplementary Fig. 1 Characterization of mechanically exfoliated monolayer WS_2 samples. a Optical microscope image of a mechanically exfoliated WS_2 sample on Si/SiO_2 substrate. **b** Optical microscope image of mechanically exfoliated WS_2 sample 1 on a quartz substrate. **c** Optical microscope image of masked mechanically exfoliated WS_2 sample 1 on a quartz substrate for transient absorption spectroscopy measurement. **d** Raman spectroscopy of mechanically exfoliated WS_2 samples on Si/SiO_2 substrate confirming the monolayer.

4. Supplementary Note 4 - PL data for chemically treated monolayer WS_2

Note that the "treated sample" without specification refers to 2D WS_2 treated with the developed protocol here. The PL spectra for H-TFSI-treated WS_2 monolayers at low temperature are not stable over time (blinking).

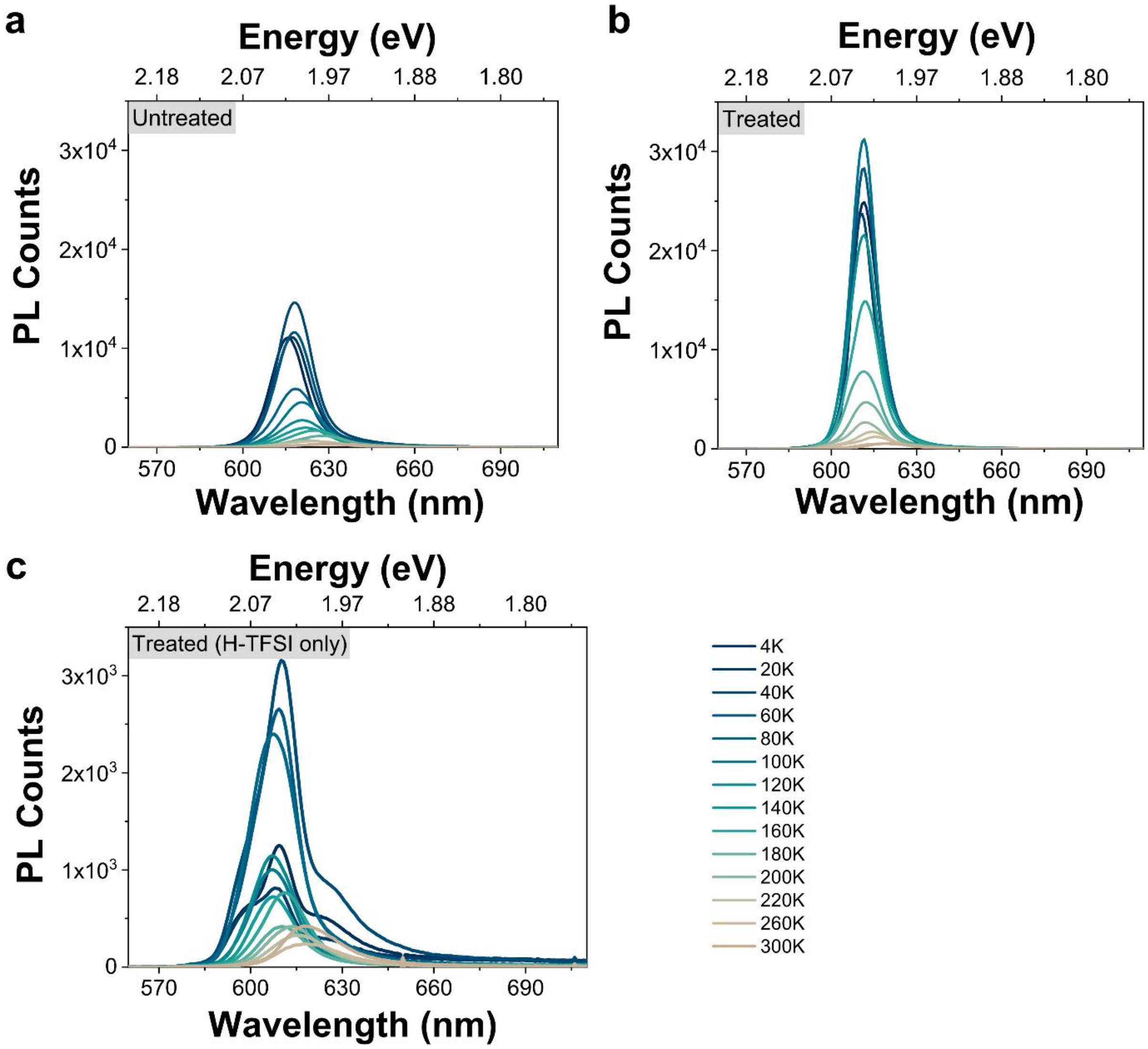

Supplementary Fig. 2 PL spectra measured for different temperatures on the untreated, H-TFSI-only treated, and treated monolayer WS_2 sample.

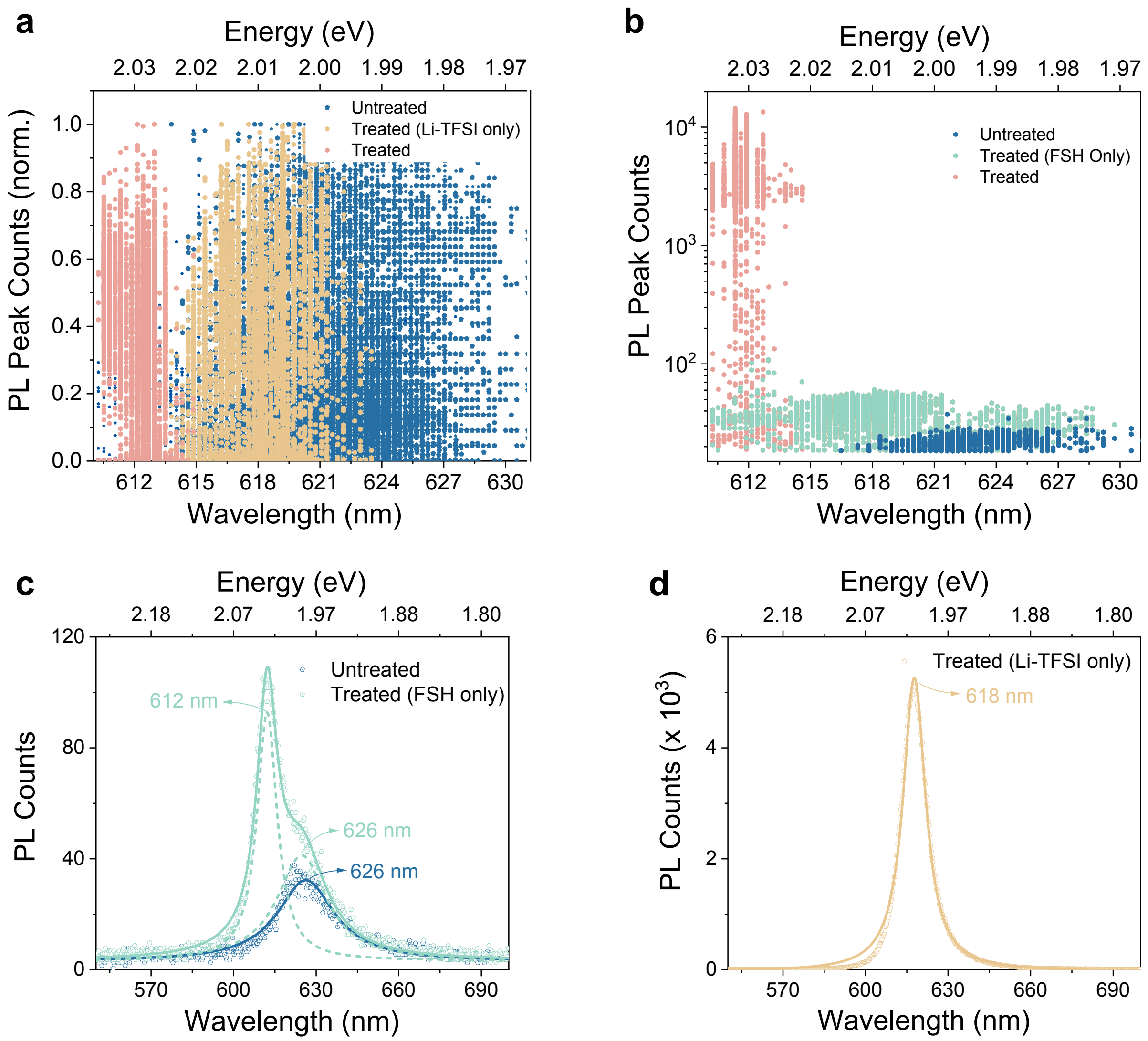

Supplementary Fig. 3 **a** Normalized PL peak counts and corresponding peak position of different samples with varied treatments. **b** Comparison of PL counts before and after treatments on the same sample. **c** Representative PL spectra for untreated and 2-furanmethanothiol (FSH)-treated monolayer WS_2. **d** Representative PL spectra for bis(trifluoromethane) sulfonimide lithium salt (Li-TFSI)-treated monolayer WS_2. The decomposed Lorentzian peak fitting is presented in dashed line and cumulative Lorentzian peak fittings are presented in solid line.

In Fig. S3 a and b, each dot signifies a PL spectrum, correlating the PL peak counts with the position of the PL maximum. The data is extracted from PL maps of WS_2 monolayers on Si-SiO_2 (90 nm), both before and after various surface treatments. Ten different untreated WS_2 monolayer flakes were mapped and normalized individually for this study (Fig. S3 a). Generally, the chemical treatments cause a blueshift in the PL spectra of the WS_2 monolayer, with narrower variations in peak positions. Notably, the chemical treatment developed in this study causes the most significant blueshift in the PL spectra of the WS_2 monolayer, which is 20 meV more compared to treatments with H-TFSI or Li-TFSI alone. The PL peaks of treated WS_2 monolayers stabilize at 2.026 eV (612 nm) with variations around 10 meV. These findings

suggest that the implemented treatment protocol enhances the p-doping effect and potentially minimizes defect occurrences, which are further supported by the charge mobility measurements, transient absorption spectroscopy measurements and DFT simulations. The inhomogeneous PL enhancement of treated 2D WS_2 can be attributed to a small percentage of other defect types such as oxygen substitutions or strain effect due to the slight roughness of the substrate surface.

The representative PL spectra of WS_2 monolayers, subjected to different treatments, are depicted in Fig. S3 c and d. The untreated WS_2 monolayers exhibit a dominant trion emission, peaking at 1.981 eV (626 nm). In contrast, the Li-TFSI-only treated sample shows a blue-shift in emission to 2.006 eV (618 nm). These results are consistent with our previous study.[9] The average treated PL intensity developed in this study is twice as high as that of Li-TFSI-only treatment.

The term 'FSH treatment' refers to the process wherein the WS_2 monolayer on a Si/SiO_2 substrate is immersed in a 0.01M FSH/Methanol solution for a duration of 6 hours. Subsequently, the sample is washed by immersion in a Methanol solvent for 48 hours, during which the solvent is periodically replaced. The FSH treatment results in a marginal enhancement of the PL intensity of the WS_2 monolayer. It is evident that the trion contribution remains strong, corresponding to the emission at 1.98 eV. However, a distinct excitonic emission peak emerges at 2.026 eV (612 nm), which suggests that more neutral excitons are populated and there is a suppression of non-radiative recombination. This peak is more blue-shifted compared to the Li-TFSI only treated sample and aligns with the peak position observed in the treatment protocol developed in this study. This indicates that the FSH treatment has contributed to defect passivation.

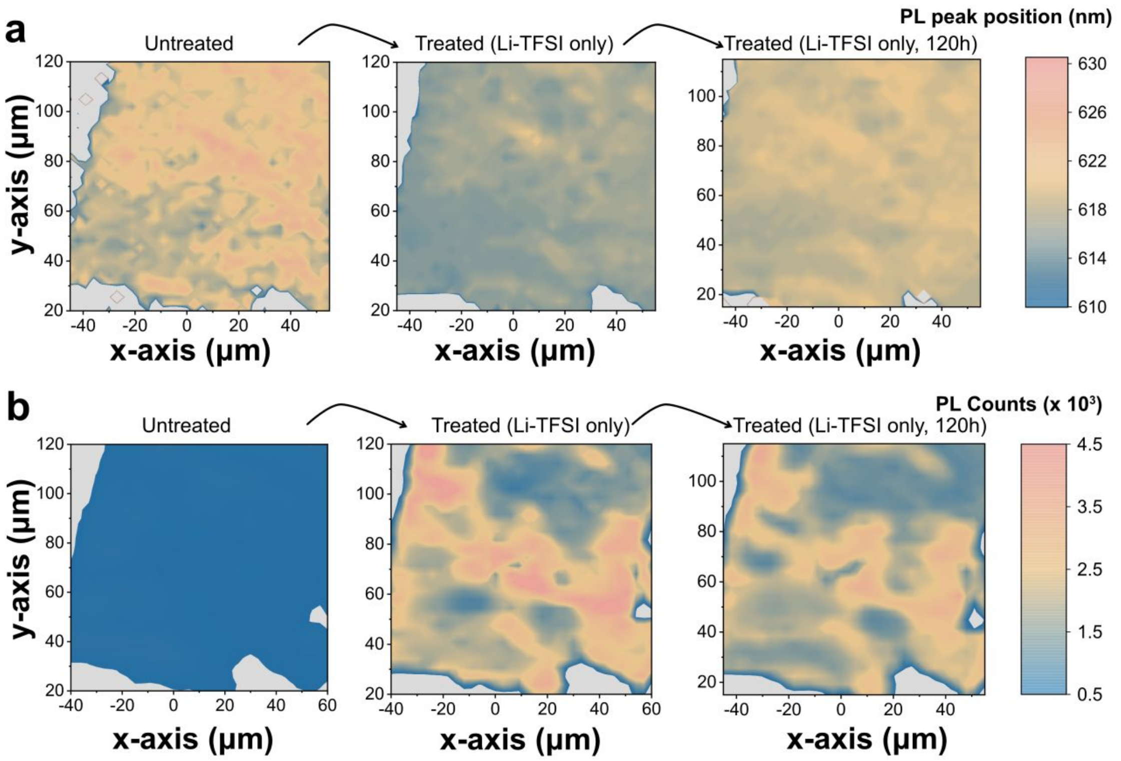

Supplementary Fig. 4 PL mapping of **a** PL peak position and **b** PL intensity change before and after the Li-TFSI only treatment on the same monolayer WS_2 sample. The PL spectra of the Li-TFSI treated sample undergo a redshift from 2.016 eV (615 nm) over time while stored in air, stabilizing at 2.003 eV (619 nm) with a position variation of 13 meV. This peak position shift is homogenous over the monolayer flake to a large extent.

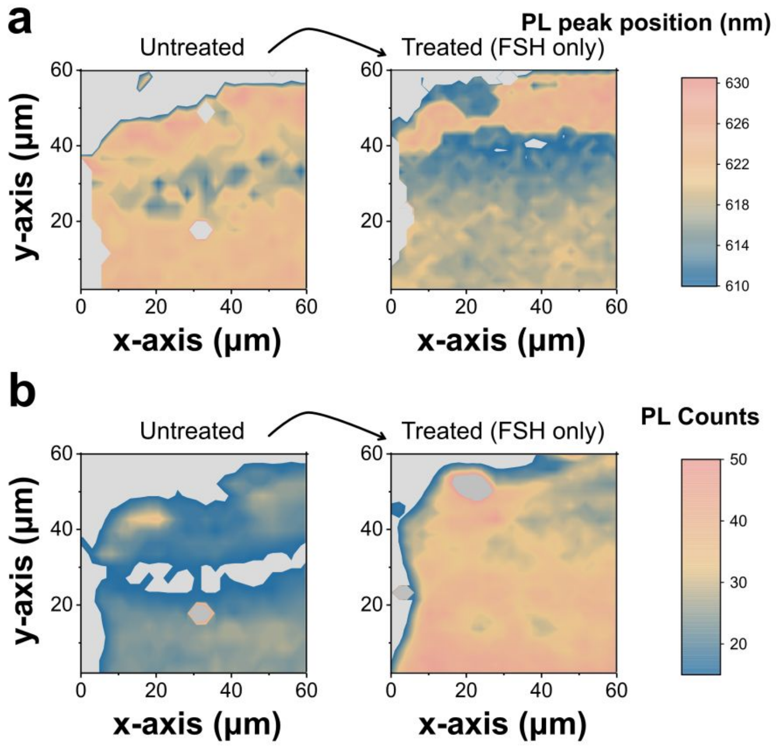

Supplementary Fig. 5 PL mapping of the PL peak position and PL intensity before and after the FSH treatment on the same monolayer WS_2 flake (sample 1).

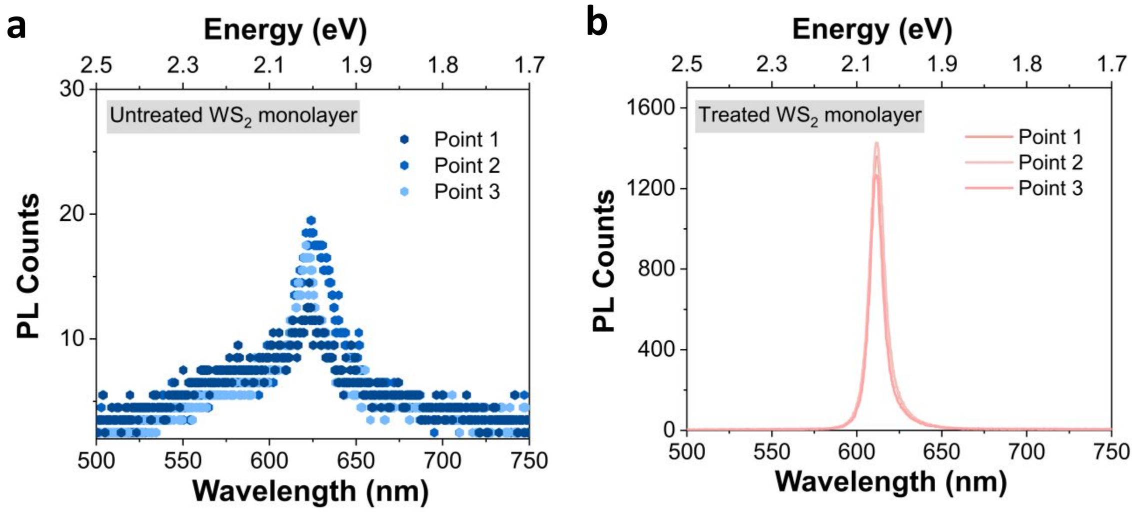

Supplementary Fig. 6 Representative PL spectra for untreated and treated monolayer WS_2 prepared by conventional scotch tape method.

To compare the 2D monolayer quality and effect of our SSCP protocol between gold-mediated mechanical exfoliation and conventional scotch tape exfoliation, we performed the chemical treatments on WS_2 monolayers prepared by the conventional scotch tape method and measured the PL of the same monolayer flake before and after the treatment at three different positions. Since the monolayer size is quite small (diameter around 10 μm), the PL mapping measurements are not performed. We find that the PL intensity of untreated 2D WS_2 samples is very similar in both exfoliation methods, and our SSCP protocol leads to significant PL enhancement and large blue shift, which is in line with our previous study on 2D MoS_2.[10]

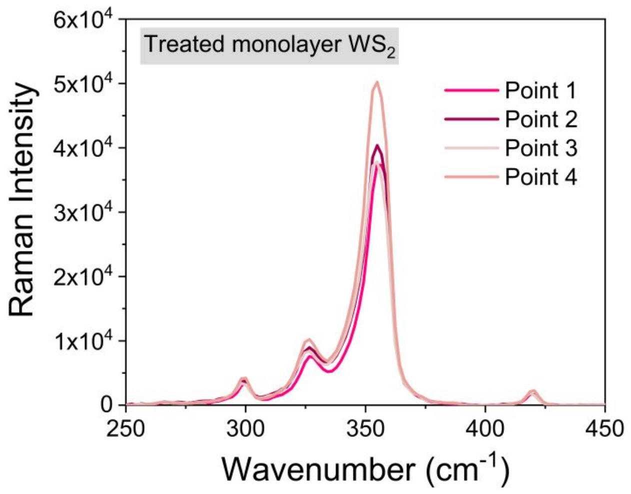

Supplementary Fig. 7 Raman spectroscopy of mechanically exfoliated WS_2 samples on Si/SiO_2 substrate which is stored in a nitrogen-filled glovebox over 12 months.

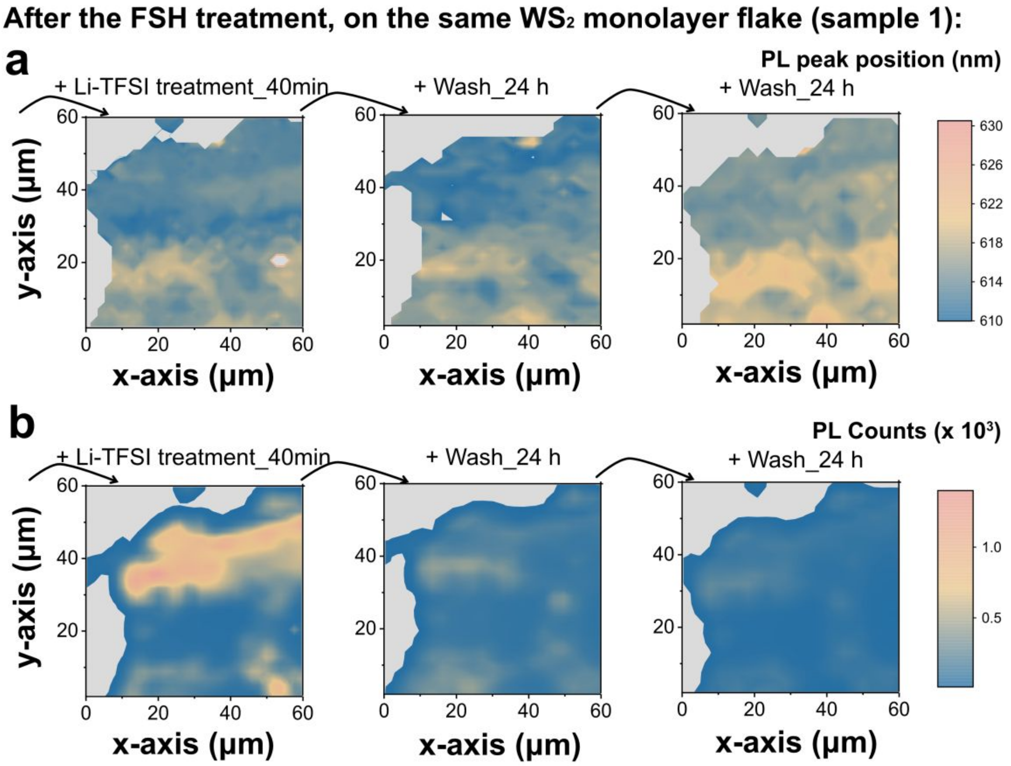

Supplementary Fig. 8 PL mapping of the PL peak position and PL intensity change after the chemical treatment and wash on the same monolayer WS_2 flake (sample 1).

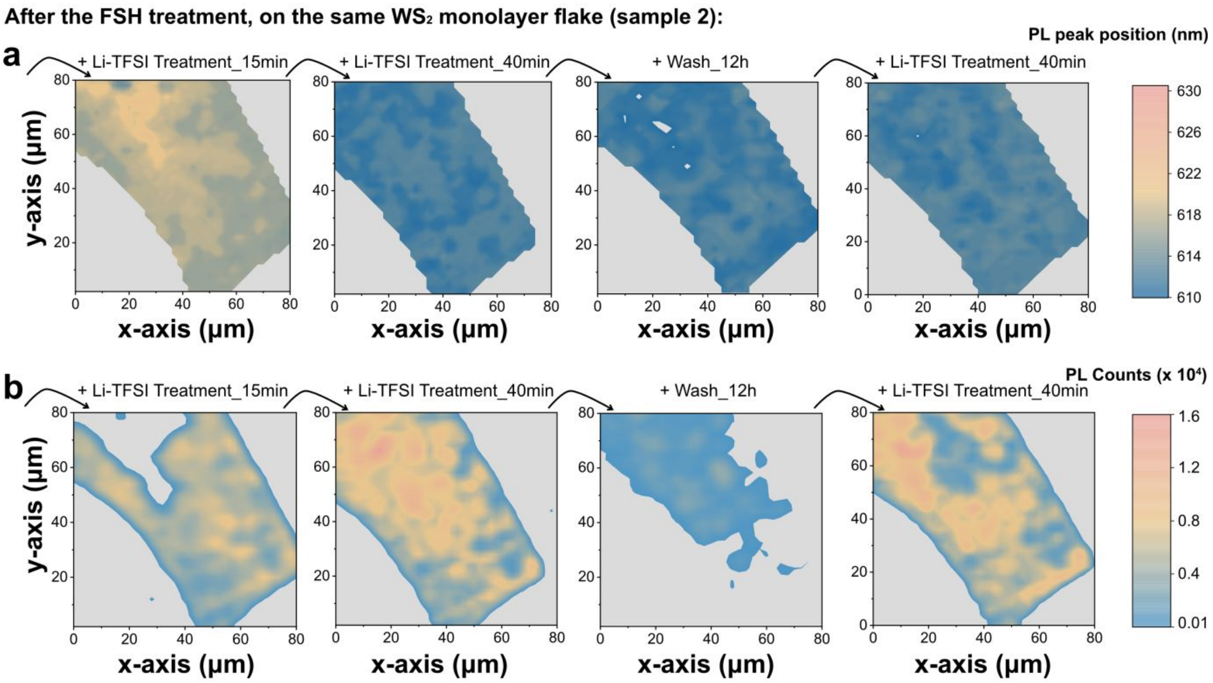

Supplementary Fig. 9 PL mapping of the PL peak position and PL intensity change during the subsequent Li-TFSI chemical treatments on the same monolayer WS_2 sample 2 after the FSH treatment.

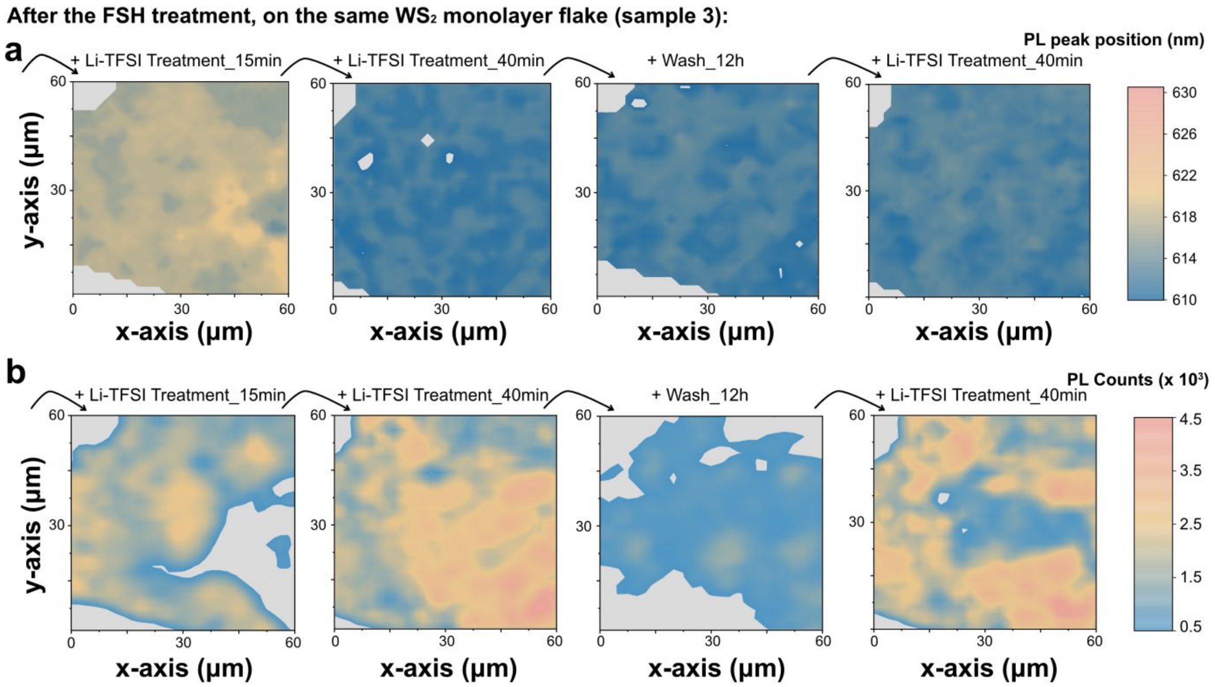

Supplementary Fig. 10 PL mapping of the PL peak position and PL intensity change during the subsequent Li-TFSI chemical treatments on the same monolayer WS_2 sample 3 after the FSH treatment.

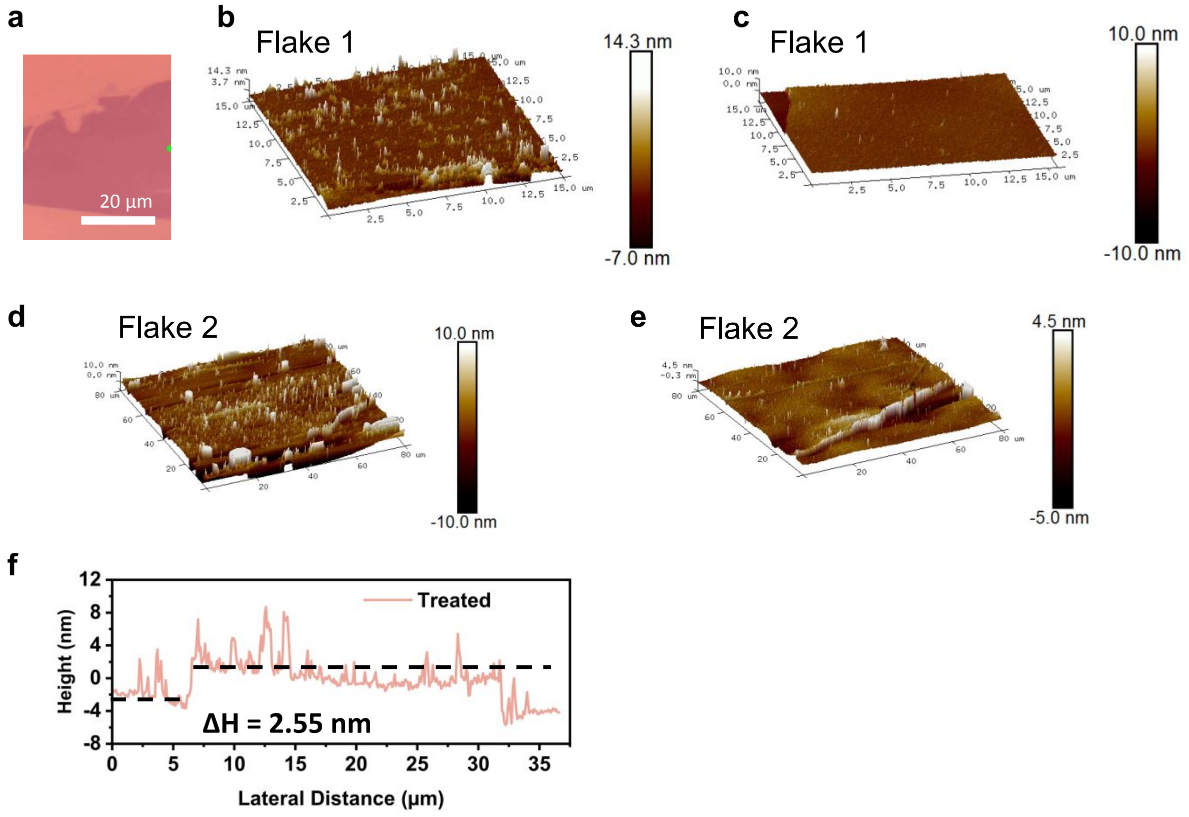

Supplementary Fig. 11 **a** Optical microscope image of a treated WS_2 monolayer. **b** AFM image of the treated WS_2 monolayer (Flake 1). **c** AFM image of the treated WS_2 monolayer after 40-min immersion in Methanol (Flake 1). **d** AFM image of the treated WS_2 monolayer

(Flake 2). **e** AFM image of the treated WS_2 monolayer after 40-min immersion in Methanol (Flake 2). **f** Average height of a treated WS_2 monolayer sample measured by AFM.

As shown in Fig. S11 and the subsequent discussion in XPS measurements, there is a large excess of Li-TFSI on the surface of the WS_2 monolayer flake. Interestingly, this large excess of Li-TFSI does not affect the doping stage since both the PL peak shift and PL peak intensity show stability after 40-min immersion in the solvent Methanol. And the PL peak shift which indicates the doping stage shows high stability even after 12h immersion in the solvent Methanol. Since our SSCP protocol involves immersion and N_2 blow drying of the films, this leads to an inhomogeneous Li-TFSI layer across the WS_2 monolayer, resulting in variable layer thickness. In the AFM height profile scan across a WS_2 monolayer, we observed a total mean thickness of the monolayer WS_2 + Li-TFSI layer ~ 2.5 nm, which suggests a typical thickness of the top Li-TFSI layer ~1.5 nm.

5. Supplementary Note 5 – FET device data

We have fabricated and characterized five untreated FET devices and six treated FET devices at V_{DS} = 1 V. The detailed parameters are presented as follows:

Supplementary Table 1. Parameters from untreated monolayer WS_2 FET devices. Mobility is extracted at V_{DS} = 1 V. The threshold voltage is exacted in the Extrapolated Linear Region (ELR). The device 2 is used in the main text.

**Device**	**L_{ch} (μm)**	**W_{ch} (μm)**	**Mobility ($cm^2/(V \cdot s)$)**	**Threshold Voltage (V)**	**Total Resistance at V_g 120 V (R_T, Ohm)**	**Total Resistance at V_g 60 V (R_T, Ohm)**
1	0.88	3.33	0.0469	75.13	15.14M	76.92M
2	1.24	4.77	0.05	74.82	10.65M	1032.09M
3	1.16	4.04	0.107	45.05	3.35M	21.2M
4	1.5	4.34	0.139	61.28	3.88M	30.39M
5	7.32	7.29	1.433	82.09	1.57M	25.19M

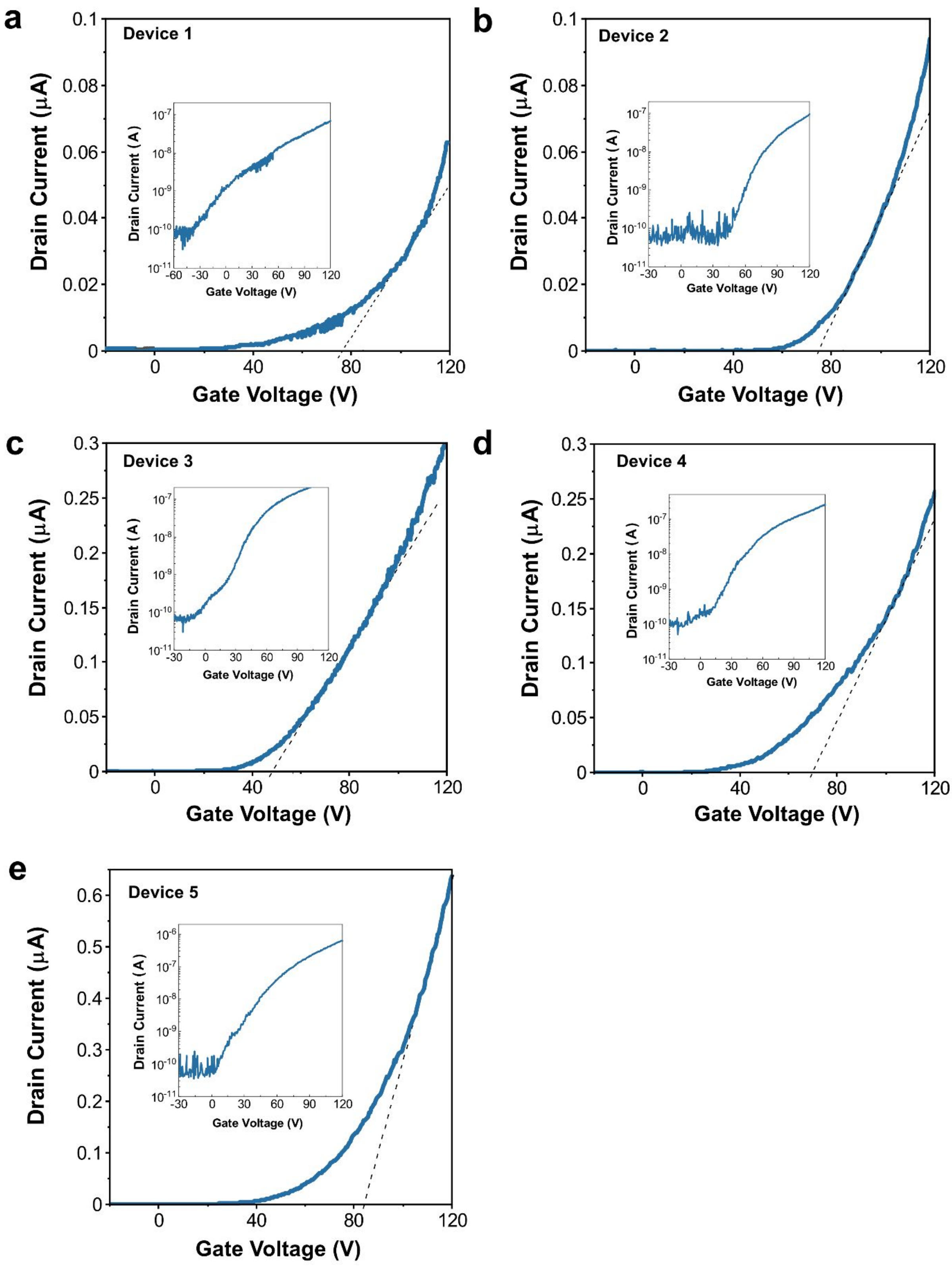

Supplementary Fig. 12 Drain current versus gate voltage of untreated monolayer WS_2 FET at drain-source voltage (V_{DS}) of 1V in linear scale. Inset: in logarithmic scale.

Supplementary Table 2. Treated devices. Mobility is extracted at V_{DS} = 1 V. The threshold voltage is exacted in the Extrapolated Linear Region (ELR). The device 6 is used in the main text.

**Device**	**L_{ch} (μm)**	**W_{ch} (μm)**	**Mobility ($cm^2/(V \cdot s)$)**	**Threshold Voltage (V)**	**Total Resistance at V_g 60 V (R_T, Ohm)**
6	2.09	16.62	69.73	3.51	9.4K
7	1.56	17.1	16.09	9.82	18.2K
8	1.15	7.59	14.85	10.62	51.3K
9	1.94	12.99	12.05	7.71	40.9K
10	0.9	10.29	10.08	20.08	29.3K
11	1.24	11.71	10.37	15.06	64.5K

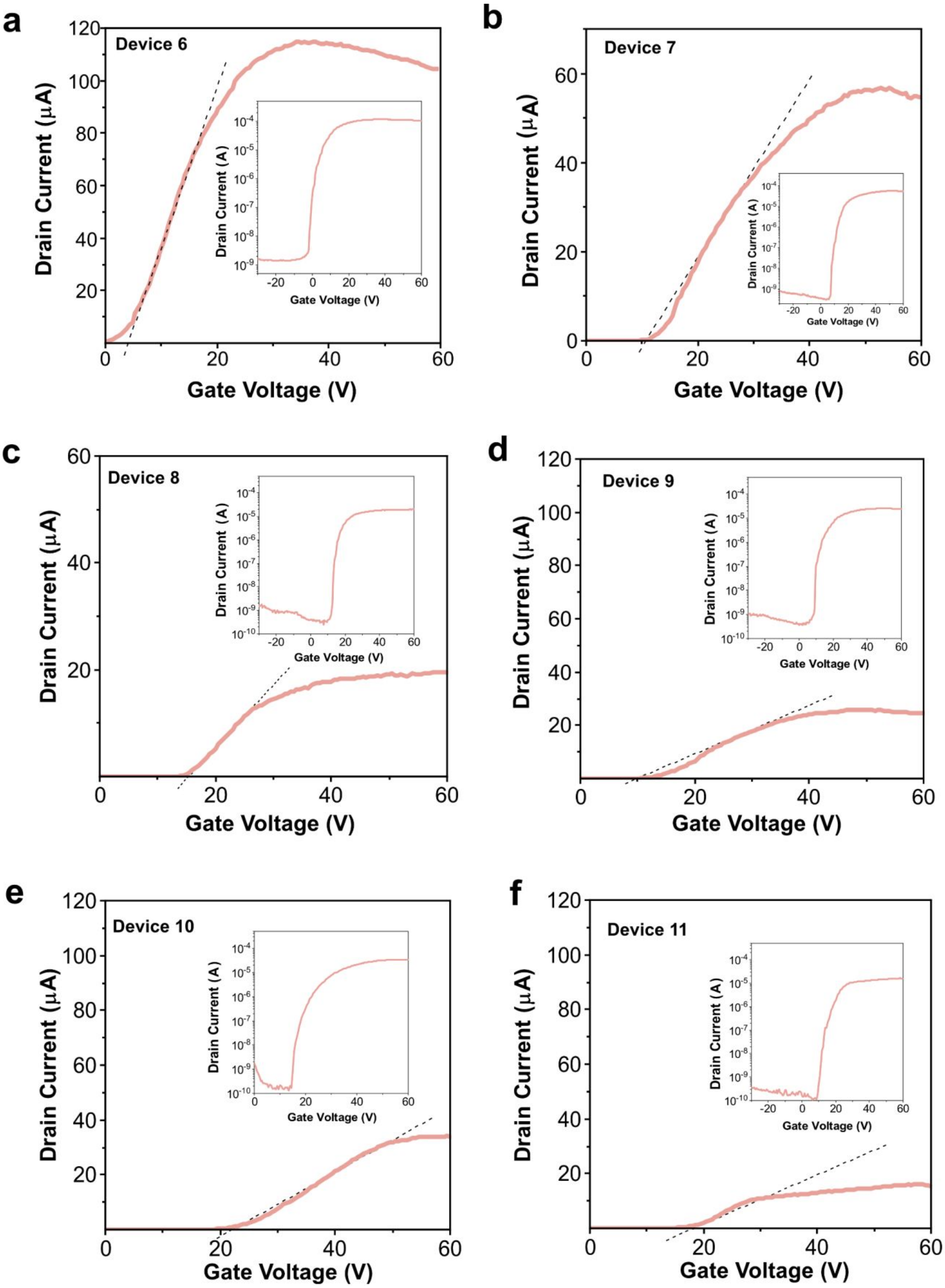

Supplementary Fig. 13 Drain current versus gate voltage of treated monolayer WS_2 FET at drain-source voltage (V_{DS}) of 1V in linear scale. Inset: in logarithmic scale.

6. Supplementary Note 6 – Transient absorption spectroscopy data

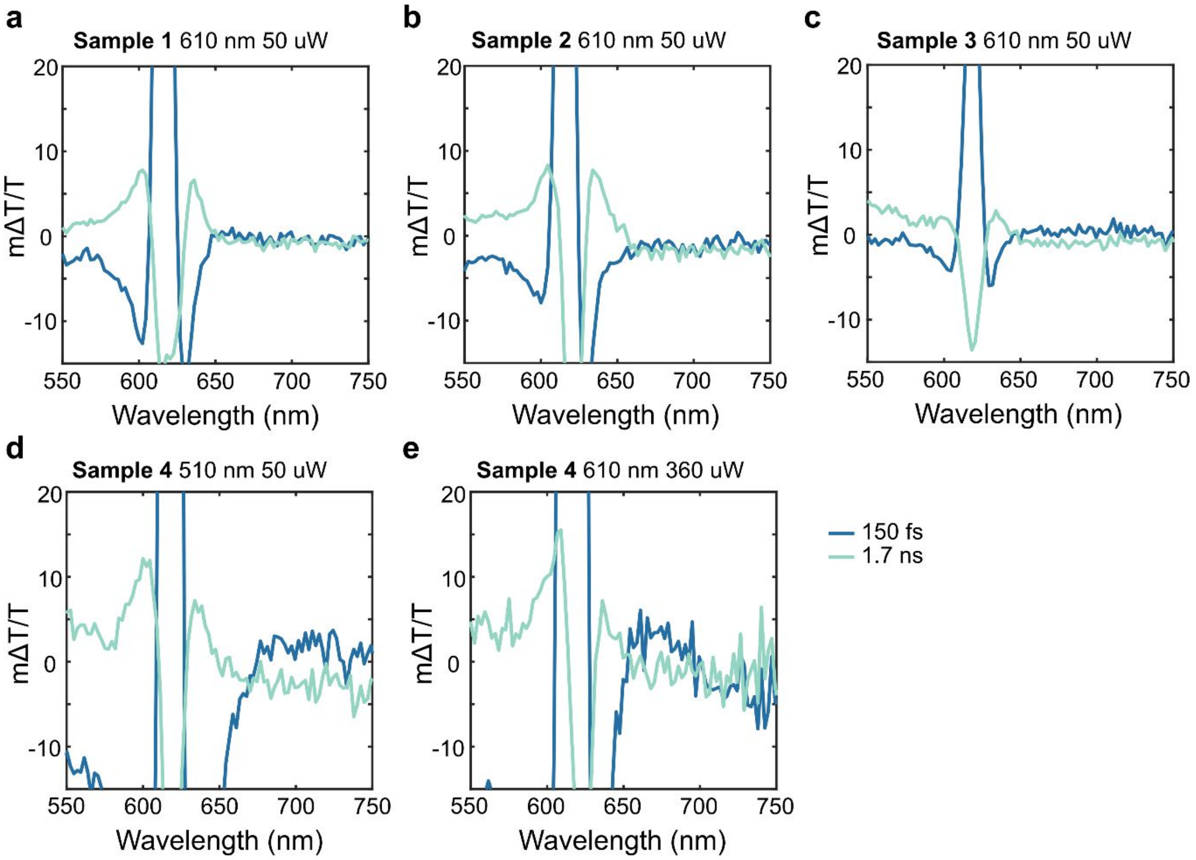

Supplementary Fig. 14 Pump-probe spectra of four untreated monolayer WS_2 samples (Sample 1 is used in the main text). Transient absorption spectra at short (150 fs) and long (1.7 ns) time delays of 4 different samples excited at 610 nm (**a**, **b**, **c**, **e**) and 510 nm (**d**) with an excitation power of 50 W (**a-d**) and 360 W (**e**).

The single value decomposition

The single value decomposition was performed using Matlab's built-in single value decomposition function [U,S,V] = svd ().

Where U is the left-singular vectors, S is a matrix containing the singular values on the diagonal, and V is the right-singular vectors.

The spectral components (P) were obtained by multiplying the left-singular vectors (U) with the square root of the singular values:

P=U*S.^.5

Similarly, the temporal evolution (T) is obtained by multiplying the square root of the singular values with the transpose of the right-singular vectors (V).

T=Ssvd.^.5*V'

By sorting the singular values in S and plotting them one can analyze the significance of each component's contribution to the total dataset. It can be seen in Figures S15a and S16a that two singular values are significantly larger than the rest for the untreated samples, whereas for the

treated sample only one singular value is significantly larger. Similarly, for the untreated samples Figures S15b, c and S16b, c show that two spectral and two temporal components are above the noise level, in line with the two large singular values that were observed. For the treated sample on the other hand, in Figure S18, only one large singular value is observed and there is also only one significant spectral and temporal component above the noise level.

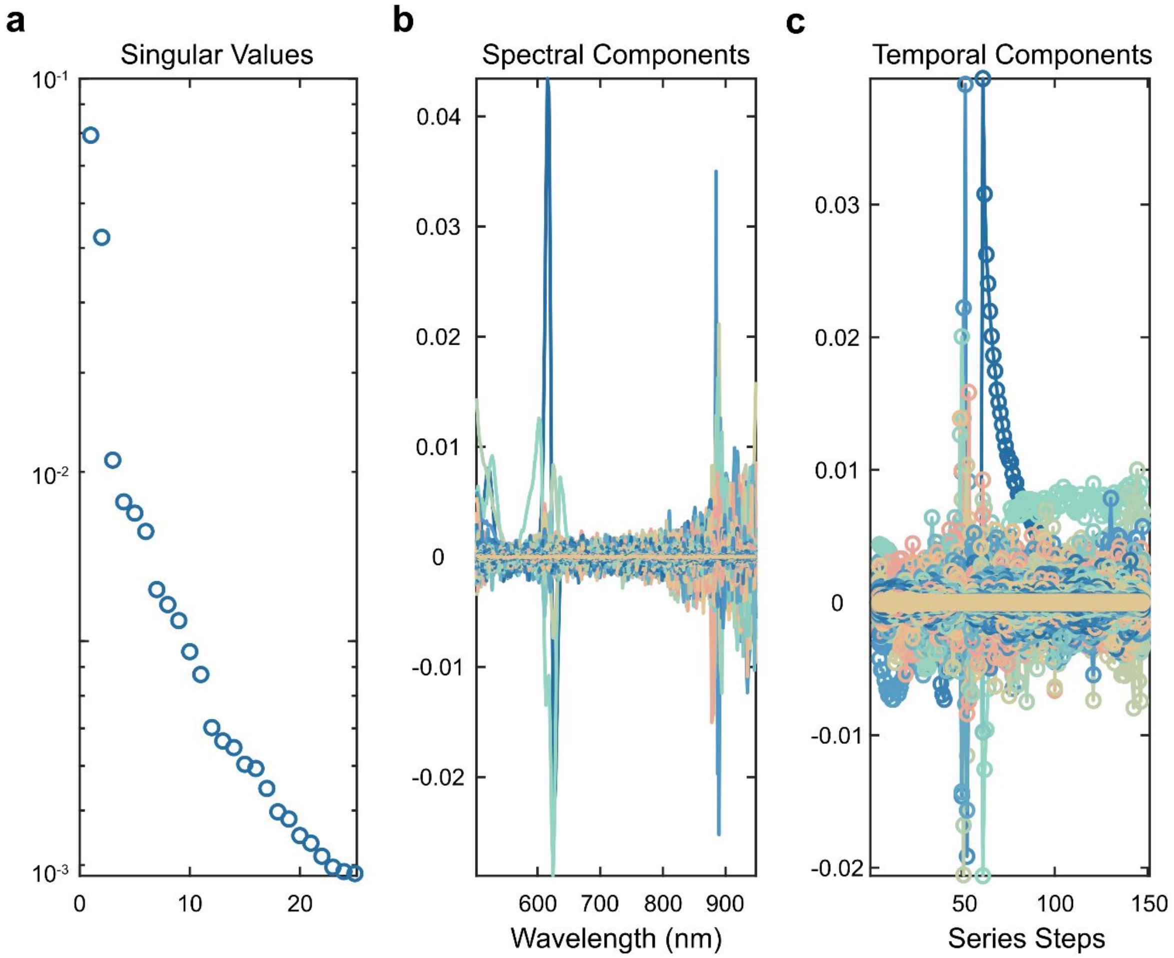

Supplementary Fig. 15 Single value decomposition of transient absorption data for sample 1 untreated. **a** Singular value for the components. **b** Spectral components. **c** temporal profiles extracted by SVD. Two components have significantly larger singular values as well as spectral and temporal features above the noise level (blue and green spectra).

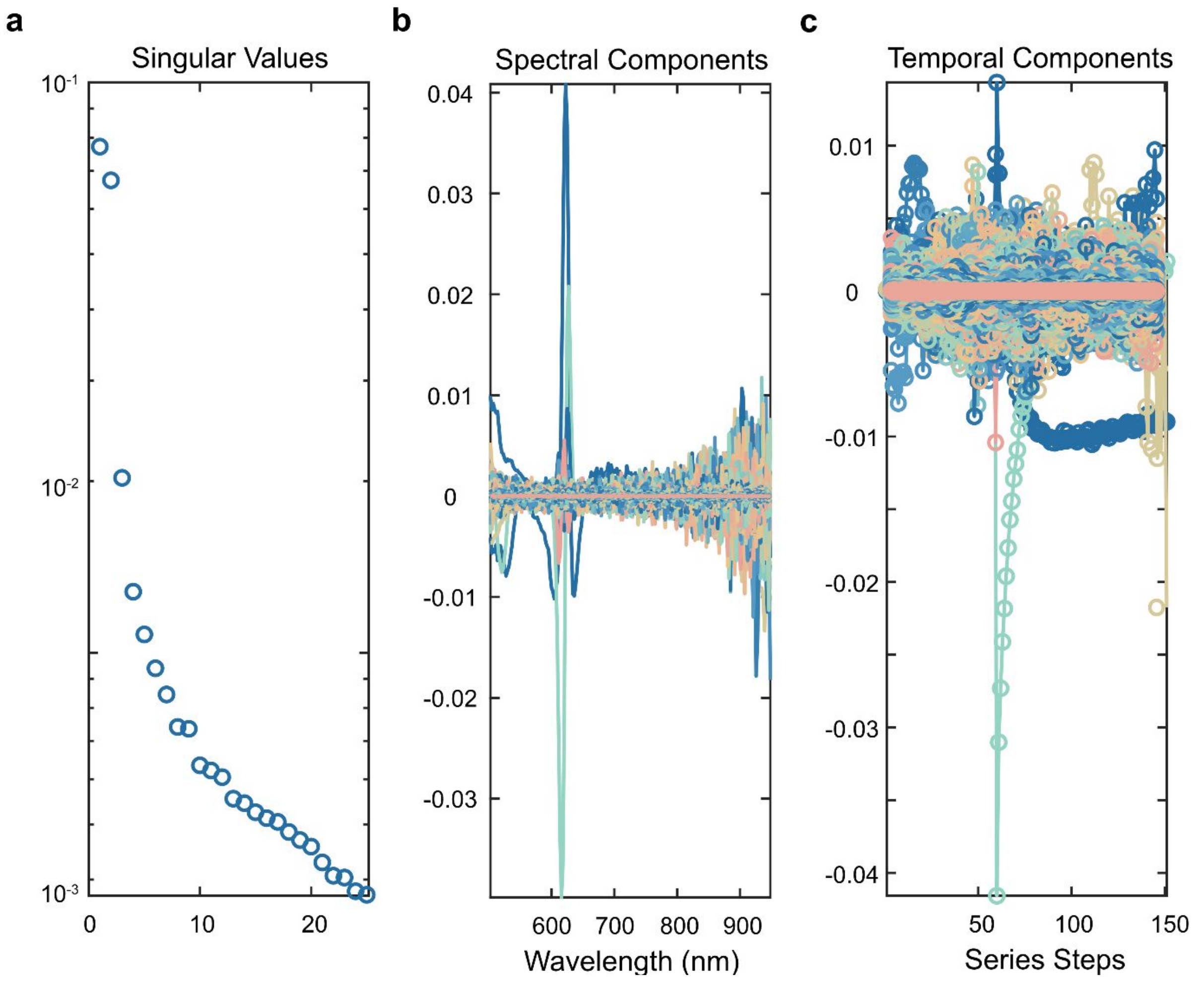

Supplementary Fig. 16 Single value decomposition of transient absorption data for sample 2 untreated. **a** Singular value for the components. **b** Spectral components. **c** temporal profiles extracted by SVD. Two components have significantly larger singular values as well as spectral and temporal features above the noise level (blue and green spectra).

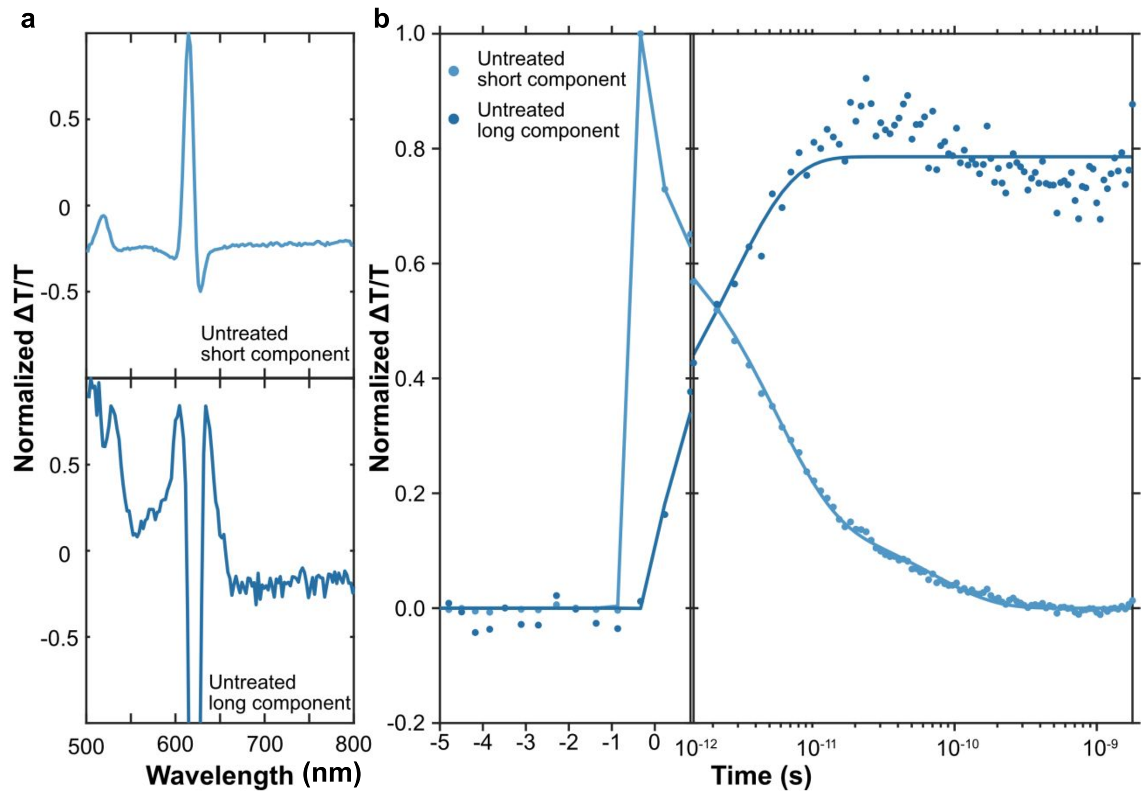

Supplementary Fig. 17 Pump-probe spectra of untreated monolayer WS_2 sample 2. a Normalized pump-probe spectra at 150 fs and 1.7 ns. **b** Kinetic profiles for the corresponding spectra in **a** and multi-exponential fits using a 185-fs-wide gaussian response function. The fitting is presented in solid lines.

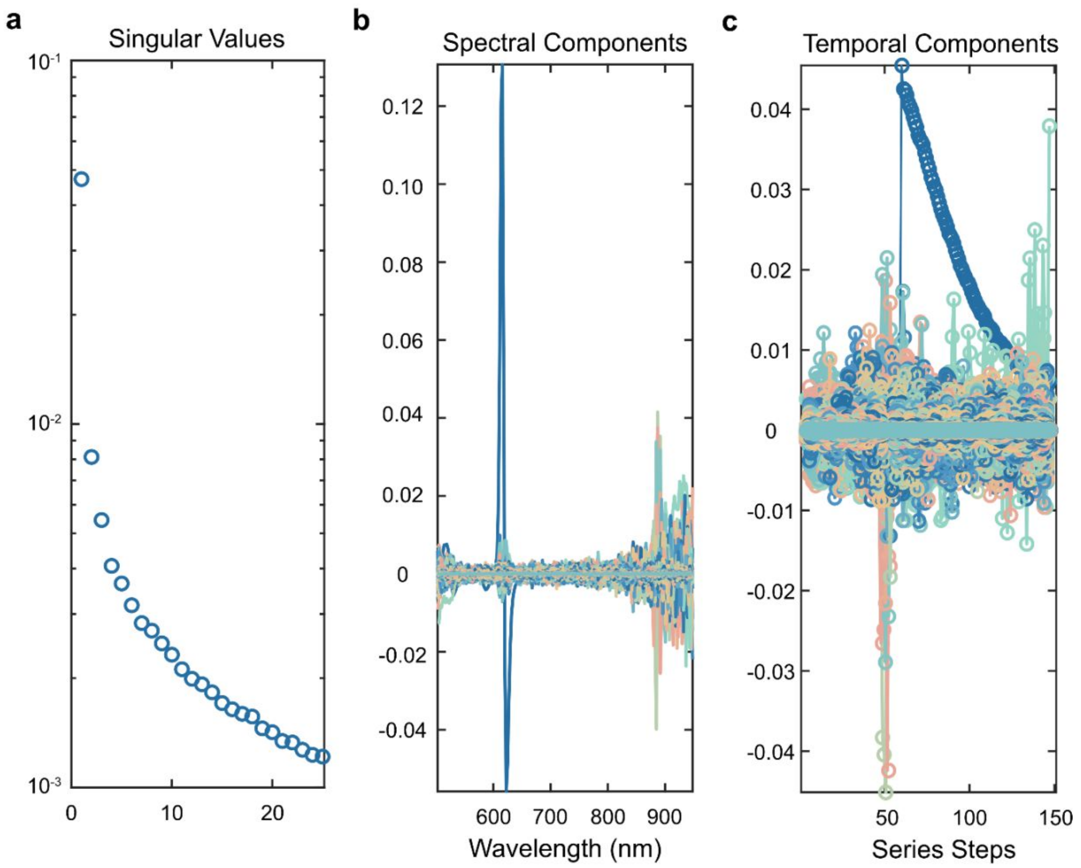

Supplementary Fig. 18 Single value decomposition of transient absorption data for sample 1 treated. **a** Singular values for the components. Spectral components (**b**) and temporal profiles (**c**) extracted by SVD. There is only 1 component with a significantly larger singular value as well as only one spectral and temporal feature above the noise level (blue spectra).

Extracting kinetic profiles for the identified species

From our SVD analysis, we conclude there are two excited state species in the untreated samples and one excited state species in the treated samples. We assign spectra to each species in the following way: For the initially excited species in the untreated samples, as well as the main species in the treated sample the average of the recorded spectra between 100-600 fs is used. For the second species in the untreated samples, the spectra between 0.8-1.8 ns are averaged. The kinetic profile associated with each spectral feature is then obtained in a least-square manner. For example, for the untreated samples, the two spectra were combined as columns in a matrix **A**, where each row contains the intensity value at a corresponding wavelength, and the columns correspond to the first and second species, respectively. The kinetic profile associated with each spectra (K) is then obtained by taking the pseudoinverse of matrix **A** times a matrix **D** containing all the experimental TA data. The calculations were carried out in MATLAB® using the pinv function:

K = pinv(A)*D

Similarly, the kinetic profile for the single component in the treated sample was extracted in the same way, but with matrix **A** only containing one spectrum.

Fitting of the Kinetic Profiles

The obtained kinetic traces were fit with multi-exponential decays using a deconvolution fitting procedure to account for the Gaussian response function at early times. The multiexponential fitting function can be described by the following equation:

$$I(t) = \sum_i A_i 0.5\ e^{0.5\left(\frac{\sigma}{\tau_i}\right)^2}\ e^{-\frac{t-t_0}{\tau_i}}\ erfc\left(\frac{1}{\sqrt{2}}\left(\frac{\sigma}{\tau_i}\right) - \frac{t-t_0}{\sigma}\right) \quad \text{(S1)}$$

Where σ is the full-width at half max (FWHM) of the Gaussian response function, t_0 is the center of the Gaussian response function τ_i is the lifetime of the ith decay and A_i is the pre-exponential factor for the ith decay. Fitting was done using a home written MATLAB® script where the experimentally obtained kinetic profile was compared to the analytically described function in Equation S1 where the lifetimes were optimized to minimize the difference using the fminsearchbnd function developed by John D'Errico (2023), MATLAB Central File Exchange.[11] The pre-exponential factors were obtained through linear regression as part of the minimization procedure. The obtained fitting parameters are summarized in Table S1 below.

Supplementary Table 3. Fitting parameters obtained from the fitting of the kinetic profiles.

Parameter	Treated	Untreated Sample 1 First component	Untreated Sample 1 Second component	Untreated Sample 2 First component	Untreated Sample 2 Second component
σ	185 fs	185 fs	185 fs	185 fs	185 fs
t_0	-275 fs	-275 fs	-275 fs	-275 fs	-275 fs
τ_1	15 fs	89 fs	15 fs	46 fs	820 fs
A_1	22.3	5.3	-1.2	8.6	-0.44
τ_2	20.5 ps	7.0 ps	2.6 ps	5.3 ps	6.0 ps
A_2	0.34	0.51	-0.9	0.58	-0.53
τ_3	128 ps	133 ps	17.9 ps	71.0 ps	91 ps
A_3	0.37	0.18	0.16	0.16	0.16
τ_4	3.2 ns	-	300 ns (fixed)	-	300 ns (fixed)
A_4	0.21	-	0.76	-	0.75

$\tau_{average}$	31.5 ps	4.7 ps	-	1.6 ps	-

7. Supplementary Note 7 – Photoelectron spectroscopy data

For the treated sample and the sample treated only with Li-TFSI, the S/W ratio could not be determined as the feature originating from WS_2 was not detected in the S2p spectra (Fig. 4). It should be noted that the feature at binding energies around 168 eV originates from the Li-TFSI salt and not from the underlying WS_2 monolayer.[12,13]

As shown in Fig. 4a, the W4f core level spectrum of the untreated WS_2 is dominated by a peak with binding energy 32.5 eV, close to reference values of monolayer WS_2.[14–16] This feature is found at similar binding energies in the three treated samples, but the relative intensity of this feature differs between the different surface treatments. After FSH treatment, the relative intensity of the WS_2 feature increases slightly (from 91.2% to 92.3%). The opposite is found for the Li-TFSI and FSH + Li-TFSI treated samples, where this feature makes up only 42.1% and 24.3% respectively. This suggests that there is a strong interaction between the W and Li-TFSI. The peak at higher binding energies, found at 34.8 eV for the untreated sample, corresponds to oxygen bonds and is found in all samples to different degrees.[17] The W5p core level is visible at 38.2 eV for the untreated sample. Due to the ionization cross section, this peak is relatively larger than W4f in HAXPES spectra compared to XPS spectra. This contrast change is a cross section effect where p-orbitals cross sections decrease slower with increasing photon energy compared to f-orbitals.[18] The addition of metallic tungsten is observed in both the Li-TFSI and FSH + Li-TFSI treated samples, at relatively higher levels in the former.[15] The formation of metallic tungsten was previously attributed to damages to the WS_2 monolayer caused by sputter cleaning with Ar^+ ions. Given that the experimental conditions are identical for all untreated and treated samples, and that this metallic feature appears exclusively in the Li-TFSI and FSH + Li-TFSI treated samples (with a higher percentage in the latter treatment), this strongly suggests that the presence of metallic tungsten is due to Li-W bonding. This peak is not distinguishable in the HAXPES measurement of the Li-TFSI treated sample, suggesting this effect occurs mainly on the immediate surface as the XPS measurements are more surface sensitive. The information depth of spectroscopic measurements increases with a factor of approximately 5 when the photon energy increases from 1486.6 eV (XPS) to 9252.8 eV (HAXPES).[19,20] We observed some uneven areas in the PL enhancement after treatment, potentially caused by oxygen defects[21] or localized strain[22], which may affect the local bonding and PL. Since our treatment protocol led to significant enhancement in both optical and electronic properties, the small amount of oxygen substitution is unlikely to play a major role here.

As shown in Fig. 4b, the second feature in the S2p spectra for these samples presents larger differences in binding energy, specifically at 169.1 eV (2$p_{3/2}$ component) for the Li-TFSI only

treated sample and at 169.6 eV ($2p_{3/2}$ component) for the FSH + Li-TFSI treated samples. No signal from the underlying WS_2 is detected in the sulfur 2*p* spectra of these two samples when using the Al-Kα source in XPS measurements. However, in the S1*s* HAXPES spectrum of the Li-TFSI treated sample (Fig. 4c), spectral features from both the Li-TFSI and the WS_2 substrate are identified. This suggests that with a larger photon energy in the HAXPES measurement, information from beneath the Li-TFSI layer can be obtained due to the increased information depth.[19,20] The S1*s* peak originating from the WS_2 layer (2470.1 eV) is consistent across the untreated WS_2, the FSH treated, and the Li-TFSI only treated sample. This consistency implies that the sulfur in the WS_2 layer remains relatively stable following both FSH and Li-TFSI surface treatments, supporting our hypothesis. However, to confirm this stability, a comparison of the sulfur intensity between the samples would be necessary, which is not reliable due to the excess Li-TFSI on the surface from the treatment.

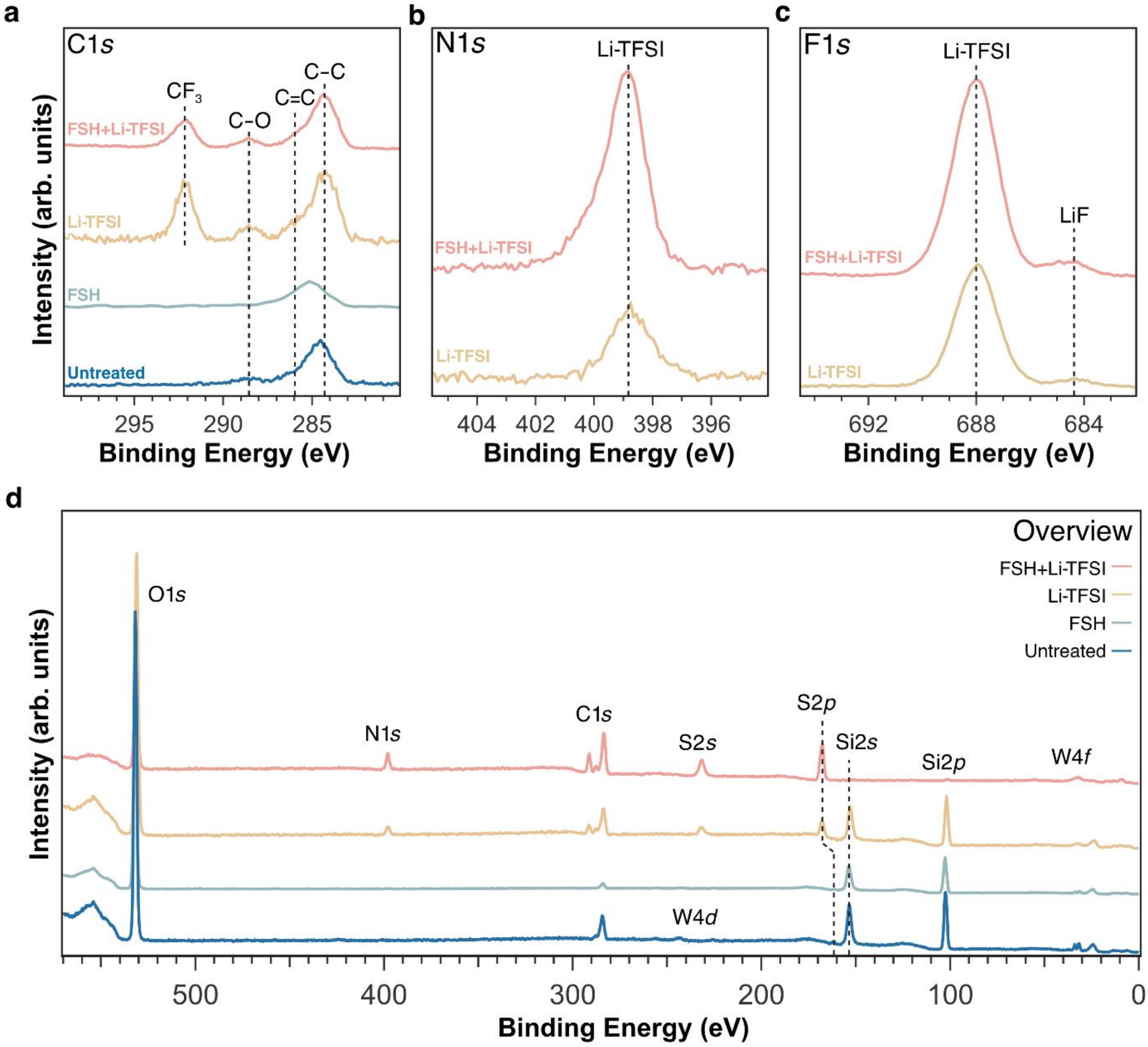

Supplementary Fig. 19 X-ray Photoelectron Spectroscopy (XPS) measurements. Core level XPS spectra in the **a** C1s, **b** N1s and **c** F1s region acquired with photon energy 1486.6 eV. **d** Overview spectra of WS_2, before and after surface treatments.

As shown in Fig. S19 carbon spectra consist of a main peak at lower binding energy that is assigned to carbon contamination (C–C/C=C) on the surface. The Li-TFSI salt contributes with a core level peak at higher binding energies (at 292 eV) seen in both the spectra for Li-TFSI and FSH + Li-TFSI, corresponding to CF_3 bonds in the salt.[12,13] It also adds to the intensity of oxygenated carbon found at 288.5 eV. Both nitrogen spectra display one main peak from the Li-TFSI salt at the same value of 398.8 eV. In the sample treated with FSH and Li-TFSI, another peak emerges as binding energy 400.1 eV which could be attributed to oxygen bonds. In the F1*s* spectra, the main corresponding to Li-TFSI is found at 688.0 eV for both samples. The smaller peak at lower binding energies is assigned to LiF and shows a small shift between the two samples.[12,23] In the Li-TFSI treated sample, this is at 684.3 eV and for the FSH + Li-TFSI treated sample it is at 684.6 eV. Both nitrogen and fluoride are found only in the Li-TFSI salt, not in the FSH salt, and the slight changes in binding energy in the N1*s* and F1*s* spectra between the two samples originate from the difference of surface below (untreated WS_2 in one case and FSH treated WS_2 in the other case).

Supplementary Table 4. Core level binding energies (eV) for WS_2 before and after surface treatment.

Core level	Untreated		FSH		Li-TFSI		FSH + Li-TFSI	
**W4*f* XPS**	**B.E. (eV)**	**%**	**B.E. (eV)**	**%**	**B.E. (eV)**	**%**	**B.E. (eV)**	**%**
W metal ($4f_{7/2}$, $4f_{5/2}$)					31.4, 33.5	17.7	31.5, 33.6	29.8
WS_2 ($4f_{7/2}$, $4f_{5/2}$)	32.5, 34.6	91.2	32.4, 34.5	92.3	32.2, 34.3	42.1	32.2, 34.3	24.3
WS_3 ($4f_{7/2}$, $4f_{5/2}$)					32.7, 34.8	31.5	32.7, 34.8	27.9
W – O ($4f_{7/2}$, $4f_{5/2}$)	34.8, 36.9	2.1	34.9, 37.0	1.3	33.7, 35.8	6.6	33.7, 35.8	16.0
WS_2 ($5p_{3/2}$)	38.2	6.7	38.2	6.4	38.2	2.1	38.5	2.0
**W4*f* HAXPES**	**B.E. (eV)**	**%**	**B.E. (eV)**	**%**	**B.E. (eV)**	**%**	**B.E. (eV)**	**%**
W metallic							31.4, 33.5	38.0
WS_2 ($4f_{7/2}$, $4f_{5/2}$)	32.5, 34.6	36.2	32.4, 34.5	35.9	32.2, 34.3	22.3	32.1, 33.2	25.4
WS_3 ($4f_{7/2}$, $4f_{5/2}$)					32.7, 34.8	15.2	33.1, 35.2	28.7

W – O (4$f_{7/2}$, 4$f_{5/2}$)	35.9, 38.0	4.3	35.6, 37.7	2.4	35.5, 37.6	3.8	34.1, 36.2	7.2
WS_2 (5$p_{3/2}$)	38.1	59.5	37.7	61.7	38.0	58.7	38.5	0.8
**S2*p***	**B.E. (eV)**		**B.E. (eV)**		**B.E. (eV)**		**B.E. (eV)**	
WS_2 (2$p_{3/2}$, 2$p_{1/2}$)	162.0, 163.1		162.0, 163.1					
Li-TFSI					168.4, 169.5<br>169.1, 170.2		168.3, 169.4<br>169.6, 170.7	
**S1*s***	**B.E. (eV)**		**B.E. (eV)**		**B.E. (eV)**		**B.E. (eV)**	
WS_2	2470.1		2470.2		2470.2			
Li-TFSI					2477.5		2478.0	

Core level	Untreated	FSH	Li-TFSI	FSH + Li-TFSI
**C1*s***	**B.E. (eV)**	**B.E. (eV)**	**B.E. (eV)**	**B.E. (eV)**
C – C	284.6	285.1	284.2	284.3
C = C	286.1		285.8	285.7
C – O	288.6		288.5	288.6
Li-TFSI (CF_3)			292.2	292.1
**N1*s***			**B.E. (eV)**	**B.E. (eV)**
Li-TFSI			398.8	398.8
Li-TFSI				400.1
**F1*s***			**B.E. (eV)**	**B.E. (eV)**
LiF			684.3	684.6
Li-TFSI			688.0	688.0

8. Supplementary Note 8 – DFT data

Adsorption of FSH vs. Methanol

Since methanol has been used as a solvent in the chemical treatment protocol, the starting point here is to check whether methanol would influence the passivation of these defects or coverage of the WS_2 monolayers. Calculations of adsorption energies of methanol *vs.* 2-furanmethanothiol (FSH) molecule considered a defect-free 2D surface, 2D surface with a neutral sulfur vacancy (SV) defect, and a negatively charged sulfur vacancy defect, since these two are the most common SV types in 2D WS_2 layers.[24] The results have shown that the adsorption of the FSH molecule ends with negative adsorption energy (exothermic reaction as has been defined) only on the non-defective WS_2 layer while methanol does not adsorb on the layers in any considered case (Table S5). The stronger probability of finding FSH on the non-defective surface of the layers is a result of the wan-der-waals interaction between this molecule and the 2D WS_2 layer. This probability is much lower for the case of methanol. Though it is likely that thiol physisorbs and cover the 2D layer, such weak interaction does not change the

electronic structure of the layer as shown in Figure S20 and S21, which cannot explain the observed PL enhancement after the chemical treatment.

Supplementary Table 5. Chemisorption and physisorption of FSH were investigated to highlight the effects of each adsorbate on the 2D WS_2 layers and defective 2D WS_2 layers.

**Scenario**		**Adsorption energy (eV)**
Physisorption	Methanol on the 2D WS_2 surface	0.60
	Methanol on the neutral SV defect	0.80
	Methanol on the charged SV defect	0.73
	FSH on the 2D WS_2 surface	-0.03
	FSH on the neutral SV defect	0.03
	FSH on the charged SV defect	0.56
Chemisorption (-S-H bond break)	FSH on the neutral defect (proton forming H_2)	-0.75
	FSH on the charged defect (proton forming H_2)	-0.01
	FSH on the neutral defect (proton on WS_2 surface)	1.13
	FSH on the charged defect (proton WS_2 surface)	1.25
	FSH on the neutral defect (proton goes to a second defective case)	-1.12
	FSH on the charged defect (proton goes to a second defective case)	-0.67

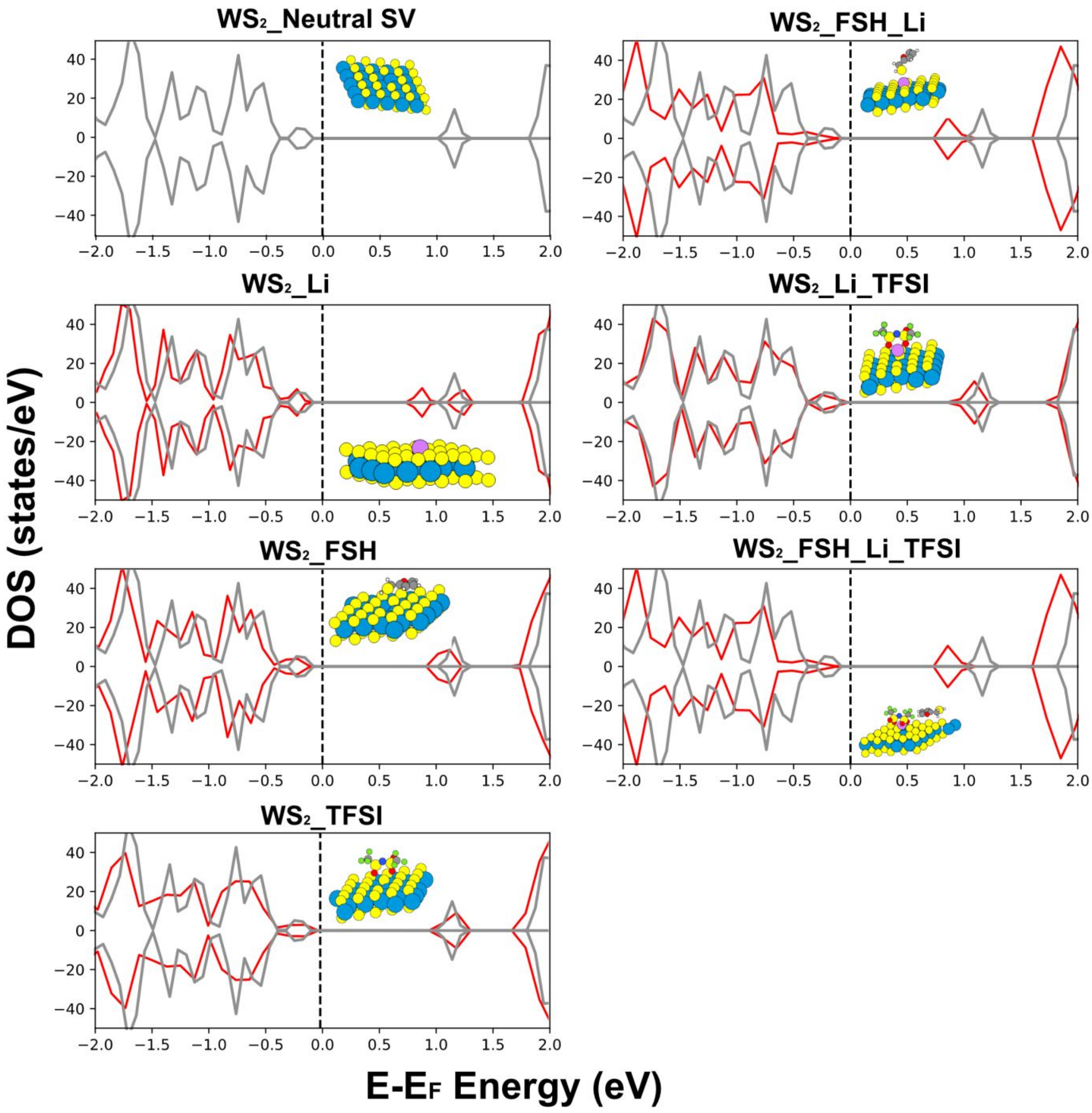

Supplementary Fig. 20 Summed PDOS on the WS_2 layer atoms for the system without adsorbates (grey) and with adsorbates (red). Here, a neutral SV defect and physical adsorption are considered.

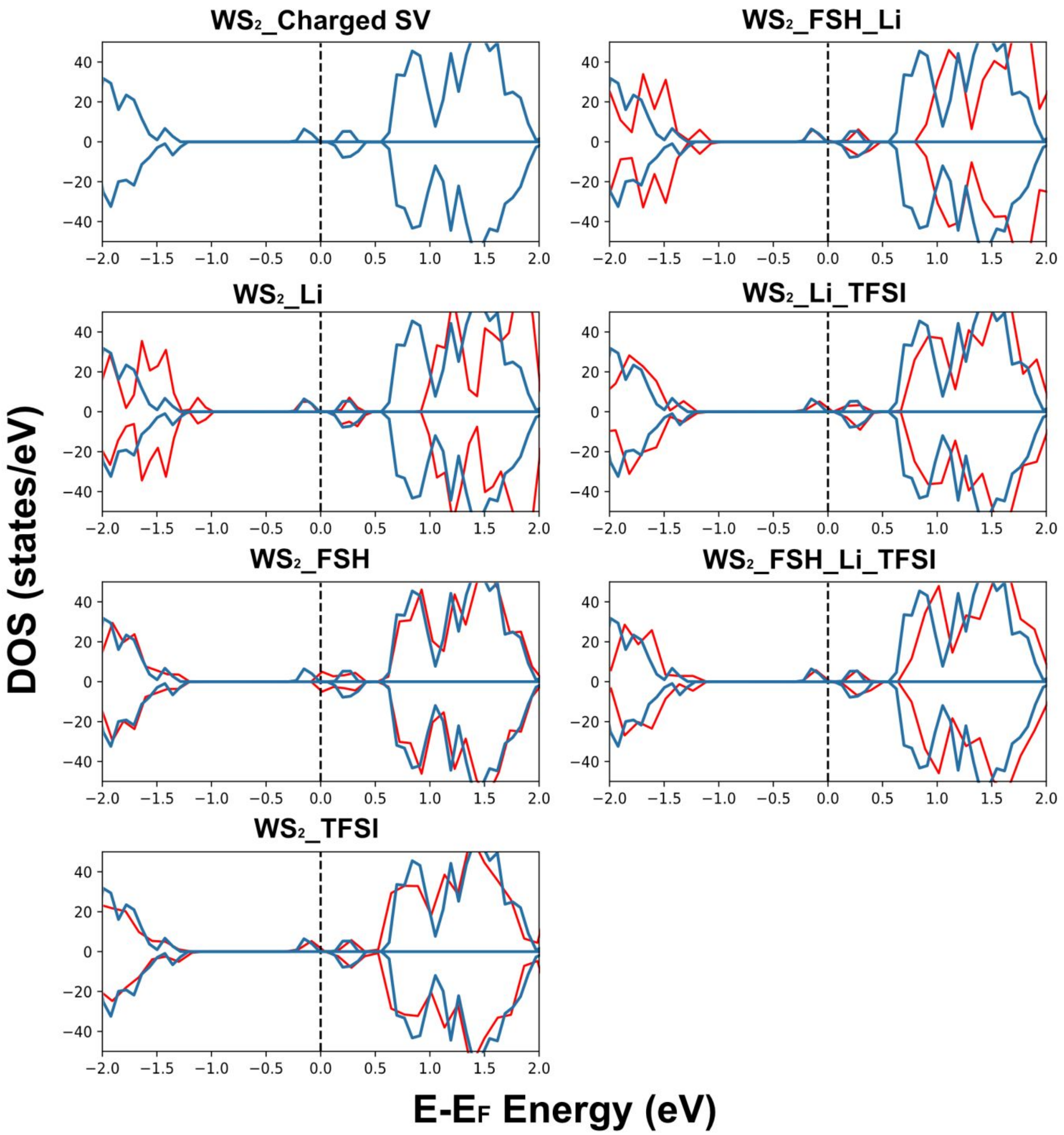

Supplementary Fig. 21 Summed PDOS on the WS_2 layer atoms for the system without adsorbates (blue) and with adsorbates (red). Here, a charged SV defect and physical adsorption are considered.

Chemical Adsorption of FSH

The bond cleavage between S and H producing a covalent bond between -S and the SV defective WS_2 monolayer is a possibility that would directly affect the electronic structure of the WS_2 2D layer. This possibility is investigated by considering three possibilities:

i) The protons from the -SH group in the FSH molecule combine and form H_2 after the cleavage;
ii) The protons from the -SH group in the FSH molecule get adsorbed on the surface of the 2D layer after the cleavage;
iii) The protons from the -SH group in the FSH molecule get adsorbed on another SV defect on the surface of the 2D WS_2 layer.

These possibilities are evaluated considering two types of 2D WS_2 layer: 2D WS_2 layer with neutral SV defects and 2D WS_2 layer with negatively charged SV defects. Clearly, the cleaved -S molecule prefers to adsorb on the neutral SV defect as compared with the negatively charged SV (see Table S5). Moreover, depending on where the lost proton adsorbs, the bond cleaved between S and H becomes thermodynamically likely. For the first scenario, where H_2 is formed, an adsorption energy of –0.75 eV is computed (Fig. S22). By bonding the thiol on the neutral SV defect and adsorbing the proton on a second sulfur defect, adsorption energy of the order of –1.12 eV is computed. Both scenarios involve exothermic reactions. On the other hand, it is unlikely that the lost proton gets adsorbed on the 2D surface according to our DFT simulation that yielded positive adsorption energies of 1.13 eV and 1.25 eV for the neutral and charged defects, respectively. From this analysis, one can infer that the bond cleavage of the thiol group would more likely lead to the formation of a newer bond between the -S molecule and the neutral SV defect with a minor chance to bond on the SV charged defect, but still the thermodynamic driving force for the -S-H cleavage depends on the local environment. Zhang *et al.* have shown via ab initio molecular dynamics (AIMD) that thiol deprotonation has a barrier of only 0.17 eV for the case of 2D MoS_2.[25] They also showed pH-dependent adsorption energy in agreement with our findings that the local environment can affect the passivation of the SV defects by controlling the chemical potential felt by the left proton. Though the electronic structure of the defective 2D layer does not change with the physisorbed FSH molecule, the formation of a new chemical bond, on the other hand, displayed an effect on the electronic structure of the 2D layer. As shown in Fig. S22, the localized defective states are split into the band gap and shifted to energies closer to the top of the conduction band with the chemical adsorption of FSH. This turns the defects shallower and less likely to trap electrons.

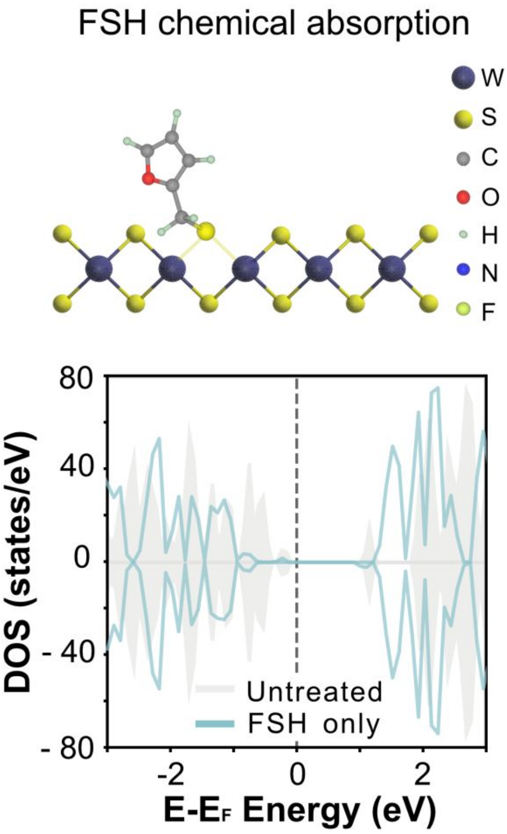

Supplementary Fig. 22 Possible mechanism for how small molecule FSH interacts with the WS_2 monolayer surface using DFT calculation.

Adsorption of Li-TFSI

To elucidate the effects associated with the developed chemical treatment protocol, we conducted a comprehensive analysis, commencing with electronic structure calculations (summed PDOS of the atomic layer) for distinct cases (Fig.S20 and S21). Our objective was to identify modifications capable of inducing changes in the layer's electronic structure, subsequently influencing the PL intensities. Subsequently, a thermodynamic analysis was employed to assess the feasibility of scenarios altering the layer's electronic structure. The only case that induced a modification in the summed PDOS of the layer was the adsorption of Li^+. Notably, for the neutral SV defects, this modification occurred exclusively when the FSH coordinates the Li^+.

Since Li^+ is found to be one of the main ones responsible for inducing changes in the 2D WS_2 electronic structure, we investigate the probabilities of finding these at the defects. Comparative energetics of Li^+ on the SV defect vs. Li^+ adsorbed on the 2D layer surface, with and without FSH coordination (the schematic pictures are shown in Figure S23) demonstrated that the presence of FSH coordinating with Li^+ stabilized the adsorption energy for both the neutral and charged SV defects involved scenarios. For the scenario involving neutral SV defects without the FSH molecule, the energy of the system where Li^+ stands on the surface of the WS_2 instead

of on the SV defect is -0.13 eV lower in energy. So, for this case, it is more likely to find Li^+ cations on the WS_2 surface than located at the SV defect. In contrast, when FSH is considered, the system with Li+ at the SV defect is -0.55 eV lower in energy than the case where Li^+ is at the WS_2 surface. Similarly, for the negatively charged SV defect, the noncoordinating case exhibited a Li^+ energy difference of – 0.44 eV, while coordination with the FSH molecule lowered it to – 0.95 eV. This energetics analysis suggests a higher likelihood of finding Li^+ on SV defective sites when the cations are coordinated by the FSH molecule (Figure 5c).

These phenomena were also predicted by ab initio molecular dynamics (AIMD) simulations using VASP (Video S1 neutral defect and S2 with charged defect). These calculations were carried out with a single-centered k-mesh and a cut-off of 400 eV. A time step of 0.75 fs was employed, resulting in a sample equilibration of 30 ps for further analysis. The temperature was set to 400 K in the canonical ensemble (fixed particle number, volume, and temperature, NVT), and a Nose-Hoover thermostat was used.[26] This slightly higher temperature was chosen to accelerate the dynamic process.

Two significant points emerge:

i) In the scenario involving negatively charged defects, the presence of ions drives the electron cloud closer to Li^+, resulting in electron removal from the 2D layer, resulting in a p-doping effect.

ii) The presence of the FSH molecule facilitates the stable Li^+ adsorption on defect sites, as evidenced by comparative energy results.

We have previously confirmed the positive impact of Li-TFSI on PL intensities, attributing it to the surface p-doping which suppresses the trion formation.[9] Similarly, Zhang *et al.* demonstrated increased PL intensity with p-doping in transition metal dichalcogenides, explained by a reduction in negative carriers and subsequently lower trion formation.[27] Our findings align with these observations, highlighting a similar p-doping effect, yet with enhanced probabilities of finding Li^+ on SV defects due to the FSH coordination.

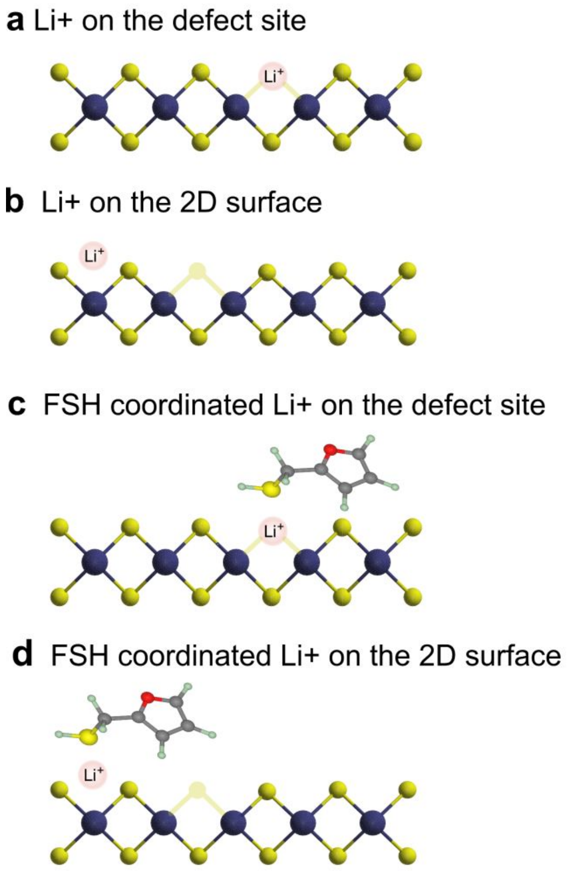

Supplementary Fig. 23 Schematic pictures of the scenarios where **a** Li+ is adsorbed on the SV defect site. **b** Li+ is adsorbed on the 2D layer surface. **c** Li+ is adsorbed on the SV defect site. with the coordination of FSH molecules. **d** Li+ is adsorbed on the 2D layer surface with the coordination of the FSH molecule.

9. References

(1) Roy, S.; Bermel, P. Electronic and Optical Properties of Ultra-Thin 2D Tungsten Disulfide for Photovoltaic Applications. *Sol. Energy Mater. Sol. Cells* **2018**, *174*, 370–379. https://doi.org/10.1016/j.solmat.2017.09.011.

(2) Patanen, M.; Aksela, S.; Urpelainen, S.; Kantia, T.; Heinäsmäki, S.; Aksela, H. Free Atom 4f Photoelectron Spectra of Au, Pb, and Bi. *J. Electron Spectros. Relat. Phenomena* **2011**, *183* (1–3), 59–63. https://doi.org/10.1016/j.elspec.2010.01.008.

(3) Shirley, D. A. High-Resolution X-Ray Photoemission Spectrum of the Valence Bands of Gold. *Phys. Rev. B* **1972**, *5* (12), 4709–4714. https://doi.org/10.1103/PhysRevB.5.4709.

(4) Kresse, G.; Furthmüller, J. Efficient Iterative Schemes for *Ab Initio* Total-Energy Calculations Using a Plane-Wave Basis Set. *Phys. Rev. B* **1996**, *54* (16), 11169–11186. https://doi.org/10.1103/PhysRevB.54.11169.

(5) Kresse, G.; Joubert, D. From Ultrasoft Pseudopotentials to the Projector Augmented-Wave Method. *Phys. Rev. B* **1999**, *59* (3), 1758–1775. https://doi.org/10.1103/PhysRevB.59.1758.

(6) Perdew, J. P.; Burke, K.; Ernzerhof, M. Generalized Gradient Approximation Made Simple. *Phys. Rev. Lett.* **1996**, *77* (18), 3865–3868. https://doi.org/10.1103/PhysRevLett.77.3865.

(7) Grimme, S.; Antony, J.; Ehrlich, S.; Krieg, H. A Consistent and Accurate *Ab Initio* Parametrization of Density Functional Dispersion Correction (DFT-D) for the 94 Elements H-Pu. *J. Chem. Phys.* **2010**, *132* (15), 154104. https://doi.org/10.1063/1.3382344.

(8) Grimme, S.; Ehrlich, S.; Goerigk, L. Effect of the Damping Function in Dispersion Corrected Density Functional Theory. *J. Comput. Chem.* **2011**, *32* (7), 1456–1465. https://doi.org/10.1002/jcc.21759.
(9) Li, Z.; Bretscher, H.; Zhang, Y.; Delport, G.; Xiao, J.; Lee, A.; Stranks, S. D.; Rao, A. Mechanistic Insight into the Chemical Treatments of Monolayer Transition Metal Disulfides for Photoluminescence Enhancement. *Nat. Commun.* **2021**, *12* (1), 6044. https://doi.org/10.1038/s41467-021-26340-6.
(10) Bretscher, H.; Li, Z.; Xiao, J.; Qiu, D. Y.; Refaely-Abramson, S.; Alexander-Webber, J. A.; Tanoh, A.; Fan, Y.; Delport, G.; Williams, C. A.; Stranks, S. D.; Hofmann, S.; Neaton, J. B.; Louie, S. G.; Rao, A. Rational Passivation of Sulfur Vacancy Defects in Two-Dimensional Transition Metal Dichalcogenides. *ACS Nano* **2021**, *15* (5), 8780–8789. https://doi.org/10.1021/acsnano.1c01220.
(11) John D'Errico. *fminsearchbnd, fminsearchcon*.
(12) Dedryvère, R.; Leroy, S.; Martinez, H.; Blanchard, F.; Lemordant, D.; Gonbeau, D. XPS Valence Characterization of Lithium Salts as a Tool to Study Electrode/Electrolyte Interfaces of Li-Ion Batteries. *J. Phys. Chem. B* **2006**, *110* (26), 12986–12992. https://doi.org/10.1021/jp061624f.
(13) Leroy, S.; Martinez, H.; Dedryvère, R.; Lemordant, D.; Gonbeau, D. Influence of the Lithium Salt Nature over the Surface Film Formation on a Graphite Electrode in Li-Ion Batteries: An XPS Study. *Appl. Surf. Sci.* **2007**, *253* (11), 4895–4905. https://doi.org/10.1016/j.apsusc.2006.10.071.
(14) Villamayor, M. M. S.; Husain, S.; Oropesa-Nuñez, R.; Johansson, F. O. L.; Lindblad, R.; Lourenço, P.; Bernard, R.; Witkowski, N.; Prévot, G.; Sorgenfrei, N. L. A. N.; Giangrisostomi, E.; Föhlisch, A.; Svedlindh, P.; Lindblad, A.; Nyberg, T. Wafer-Sized WS_2 Monolayer Deposition by Sputtering. *Nanoscale* **2022**, *14* (17), 6331–6338. https://doi.org/10.1039/D1NR08375A.
(15) Sundberg, J.; Lindblad, R.; Gorgoi, M.; Rensmo, H.; Jansson, U.; Lindblad, A. Understanding the Effects of Sputter Damage in W–S Thin Films by HAXPES. *Appl. Surf. Sci.* **2014**, *305*, 203–213. https://doi.org/10.1016/j.apsusc.2014.03.038.
(16) McCreary, K. M.; Hanbicki, A. T.; Jernigan, G. G.; Culbertson, J. C.; Jonker, B. T. Synthesis of Large-Area WS2 Monolayers with Exceptional Photoluminescence. *Sci Rep* **2016**, *6* (1), 19159. https://doi.org/10.1038/srep19159.
(17) Scarfiello, R.; Mazzotta, E.; Altamura, D.; Nobile, C.; Mastria, R.; Rella, S.; Giannini, C.; Cozzoli, P. D.; Rizzo, A.; Malitesta, C. An Insight into Chemistry and Structure of Colloidal 2D-WS2 Nanoflakes: Combined XPS and XRD Study. *Nanomaterials* **2021**, *11* (8), 1969. https://doi.org/10.3390/nano11081969.
(18) Rosmej, F. B.; Vainshtein, L. A.; Astapenko, V. A.; Lisitsa, V. S. Statistical and Quantum Photoionization Cross Sections in Plasmas: Analytical Approaches for Any Configurations Including Inner Shells. *Matter Radiat. at Extrem.* **2020**, *5* (6), 064202. https://doi.org/10.1063/5.0022751.
(19) Shinotsuka, H.; Tanuma, S.; Powell, C. J. Calculations of Electron Inelastic Mean Free Paths. XIII. Data for 14 Organic Compounds and Water over the 50 eV to 200 keV Range with the Relativistic Full Penn Algorithm. *Surf. Interface Anal.* **2022**, *54* (5), 534–560. https://doi.org/10.1002/sia.7064.
(20) Tanuma, S.; Powell, C. J.; Penn, D. R. Calculations of Electron Inelastic Mean Free Paths. IX. Data for 41 Elemental Solids over the 50 eV to 30 keV Range. *Surf. Interface Anal.* **2011**, *43* (3), 689–713. https://doi.org/10.1002/sia.3522.
(21) Barja, S.; Refaely-Abramson, S.; Schuler, B.; Qiu, D. Y.; Pulkin, A.; Wickenburg, S.; Ryu, H.; Ugeda, M. M.; Kastl, C.; Chen, C.; Hwang, C.; Schwartzberg, A.; Aloni, S.; Mo, S. K.; Frank Ogletree, D.; Crommie, M. F.; Yazyev, O. V.; Louie, S. G.; Neaton, J. B.; Weber-Bargioni, A. Identifying Substitutional Oxygen as a Prolific Point Defect in Monolayer Transition Metal Dichalcogenides. *Nat. Commun.* **2019**, *10* (1), 1–8. https://doi.org/10.1038/s41467-019-11342-2.
(22) Raja, A.; Waldecker, L.; Zipfel, J.; Cho, Y.; Brem, S.; Ziegler, J. D.; Kulig, M.; Taniguchi, T.; Watanabe, K.; Malic, E.; Heinz, T. F.; Berkelbach, T. C.; Chernikov, A. Dielectric Disorder in Two-Dimensional Materials. *Nat. Nanotechnol.* **2019**, *14* (9), 832–837. https://doi.org/10.1038/s41565-019-0520-0.
(23) Ismail, I.; Noda, A.; Nishimoto, A.; Watanabe, M. XPS Study of Lithium Surface after Contact with Lithium-Salt Doped Polymer Electrolytes. *Electrochim. Acta* **2001**, *46* (10–11), 1595–1603. https://doi.org/10.1016/S0013-4686(00)00758-1.

(24) Alaa Akkoush; Yair Litman; Mariana Rossi. A Hybrid-DFT Study of Intrinsic Point Defects in MX2 (M= Mo, W; X= S, Se) Monolayers. *Phys. status solidi a* **2024**, *221* (1), 2300180. https://doi.org/10.1002/pssa.202300180.

(25) Zhang, M.; Lihter, M.; Chen, T. H.; Macha, M.; Rayabharam, A.; Banjac, K.; Zhao, Y.; Wang, Z.; Zhang, J.; Comtet, J.; Aluru, N. R.; Lingenfelder, M.; Kis, A.; Radenovic, A. Super-Resolved Optical Mapping of Reactive Sulfur-Vacancies in Two-Dimensional Transition Metal Dichalcogenides. *ACS Nano* **2021**, *15* (4), 7168–7178. https://doi.org/10.1021/acsnano.1c00373.

(26) Daan Frenkel; Berend Smit. *Understanding Molecular Simulation From Algorithms to Applications*, Second Edition.; Academic Press, 2002.

(27) Zhang, S.; Hill, H. M.; Moudgil, K.; Richter, C. A.; Hight Walker, A. R.; Barlow, S.; Marder, S. R.; Hacker, C. A.; Pookpanratana, S. J. Controllable, Wide-Ranging n-Doping and p-Doping of Monolayer Group 6 Transition-Metal Disulfides and Diselenides. *Adv. Mater.* **2018**, *30* (36). https://doi.org/10.1002/adma.201802991.
